# A Biomimetic PLK1 Nanodelivery System Promotes Migrasome‐Mediated Mitocytosis to Restore Efferocytosis and Functional Recovery After Spinal Cord Injury

**DOI:** 10.1002/advs.78367

**Published:** 2026-10-26

**Authors:** Zhenxin Hu, Haoli Wang, Jing Huang, Junhua Zhou, Jialong Hou, Hanwei Miao, Huixin Tan, Yining Wang, Hao Hu, Na Li, Pengcheng Qiu, Hongyu Zhang, Da He, Qingqing Wang

**Affiliations:** ^1^ Department of Orthopaedic Surgery Sir Run Run Shaw Hospital Zhejiang University School of Medicine Hangzhou Zhejiang China; ^2^ Cixi Biomedical Research Institute Wenzhou Medical University Ningbo Zhejiang China; ^3^ Department of Spine Surgery Peking University Fourth School of Clinical Medicine Beijing China; ^4^ Department of Neurology The First Affiliated Hospital of Wenzhou Medical University Wenzhou Zhejiang China; ^5^ Department of Spine Surgery Beijing Jishuitan Hospital Capital Medical University Beijing China

**Keywords:** functional recovery, macrophage efferocytosis, mitocytosis, PLK1, spinal cord injury

## Abstract

Excessive accumulation and insufficient clearance of apoptotic cells following spinal cord injury (SCI) are key factors contributing to persistent injury. Macrophage efferocytosis is essential for resolving injury but is compromised by mitochondrial dysfunction and lysosomal overload. Maintaining mitochondrial functional homeostasis is therefore crucial for preserving the acidic lysosomal environment, alleviating the cellular lysosomal burden, and sustaining efferocytosis. Common mitochondrial quality control (MQC) pathways such as mitophagy increase the lysosomal burden. Here, we investigated how polo‐like kinase 1 (PLK1) regulates macrophage efferocytosis and elucidated the underlying mechanism of migrasome‐mediated mitocytosis. We demonstrate that PLK1 regulates TSPAN4‐dependent migrasome formation, which drives the selective extrusion of damaged mitochondria, alleviates the lysosomal burden, and increases efferocytic capacity. To enable targeted PLK1 delivery, we engineered macrophage membrane‐coated, reactive oxygen species (ROS)‐responsive nanocomplexes encapsulating PLK1 plasmids (MM‐NPs@PLK1). This biomimetic nanoplatform preferentially accumulates in the injured spinal cord, promotes macrophage‐associated PLK1 delivery, enhances mitocytosis and efferocytosis, supports neural tissue repair, and significantly improves locomotor recovery in SCI mice. In summary, our study reveals a PLK1‐regulated MQC mechanism that sustains macrophage efferocytosis through migrasome‐mediated mitocytosis, providing a promising therapeutic strategy for SCI.

## Graphical abstract

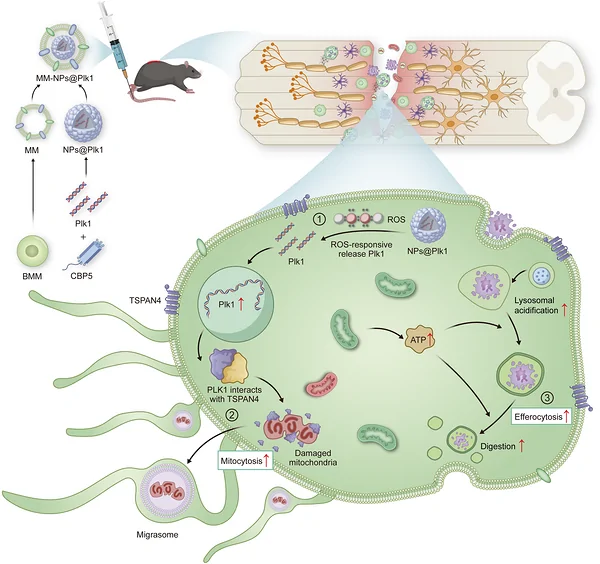

## 1 Introduction

Spinal cord injury (SCI) is a devastating neurological disease that often leads to the permanent loss of sensory, motor, and autonomic nerve functions [1]. Excessive apoptotic cells (ACs) accumulation occurs within hours to days after SCI because of prolonged oxidative stress, excitotoxicity, and cytokine storms [2]. Without timely clearance, these ACs undergo secondary necrosis, releasing damage‐associated molecular patterns (DAMPs) that subsequently trigger proinflammatory signalling pathways, cause the overproduction of proinflammatory cytokines, and exacerbate tissue damage [3]. This “cell death–inflammation” feedback loop is a major obstacle to injured spinal cord repair. Therefore, enhancing AC clearance is vital for improving recovery after SCI.

Under normal physiological conditions, the efficient phagocytosis of ACs by professional phagocytes, a process known as efferocytosis, prevents the release of intracellular contents, suppresses inflammatory signalling, maintains tissue homeostasis, and resolves inflammation [4, 5]. However, in the context of SCI, injury‐recruited macrophages migrate to the injured area and phagocytose many accumulated ACs [6]. The excessive phagocytic load imposes a heavy lysosomal burden, impairing lysosomal acidification and blocking AC degradation. Consequently, undegraded cargo releases stress signals that disrupt metabolic homeostasis in macrophages [7]. Macrophage dysfunction and death further exacerbate the AC death cascade, ultimately leading to persistent injury [8, 9]. Similar efferocytosis dysfunction has been implicated in other inflammatory diseases, such as intestinal inflammation, osteoarthritis, and rheumatoid arthritis [10, 11]; however, its contribution to SCI pathology remains poorly understood.

Efferocytosis is a highly energy‐dependent process involving multiple steps, including cytoskeletal rearrangement, phagocytic cup formation, content internalization, and phagolysosomal digestion [12, 13]. Mitochondria supply the ATP necessary for these steps through oxidative phosphorylation [13, 14], and maintaining the mitochondrial membrane potential (MMP) and clearing reactive oxygen species (ROS) are crucial for the maintenance of lysosomal acidification and membrane stability [15]. In the context of SCI, mitochondrial function is severely impaired, resulting in MMP loss, excessive ROS accumulation, and disrupted calcium homeostasis, which lead to impaired ATP synthesis and compromised efferocytosis function [16]. Damaged mitochondria are removed via canonical mitochondrial quality control (MQC) mechanisms such as mitophagy, mitochondria‐derived vesicle (MDV) activation, and the mitochondrial unfolded protein response (UPR^mt^), and these processes rely on lysosomes [17, 18, 19]. This dependence aggravates the lysosomal burden during continual efferocytosis, resulting in the formation of a destructive loop between mitochondrial injury and lysosomal dysfunction [20]. Thus, developing an alternative MQC strategy that selectively clears damaged mitochondria without imposing an additional lysosomal burden is crucial for sustaining efferocytosis after SCI.

Notably, a breakthrough in MQC research was the discovery of mitocytosis, a novel mechanism mediated by migrasomes, which are membrane‐bound organelles formed at the tips of retracting protrusions in migrating cells [21]. Migrasomes are evolutionarily conserved across multiple cell types, from fibroblasts to neurons, and participate in diverse biological processes by transferring functional molecules (e.g., proteins, RNAs, and lipids) and organelles such as mitochondria, regulating tissue morphogenesis, and facilitating wound healing [22, 23]. While cells employ several MQC strategies, these canonical pathways rely on lysosomal degradation. In the context of SCI and efferocytosis dysfunction, this dependence exacerbates lysosomal overload and hinders the rapid restoration of cellular homeostasis. In contrast, mitocytosis involves selective sequestration of damaged mitochondria (marked by low membrane potential and high ROS levels) into migrasomes that detach from the parent cell, thereby completely circumventing lysosomal degradation [21]. In vitro studies further demonstrated that mitocytosis selectively clears mitochondria with harmful mtDNA mutations and protects cells from oxidative stress [21]. Thus, these findings establish migrasome‐mediated mitocytosis as a unique, lysosome‐independent MQC mechanism that overcomes the key limitations of canonical MQC strategies in the context of efferocytosis dysfunction, making it an ideal target for restoring mitochondrial and efferocytosis function in SCI.

Migrasome biogenesis is tightly linked to cytoskeletal dynamics: actin polymerization drives the formation of retraction fibres, whereas tetraspanin proteins such as TSPAN4 stabilize migrasome membranes and mediate mitochondrial recruitment [24, 25]. Given the essential role of cytoskeletal remodelling in migrasome biogenesis and mitochondrial trafficking, kinases that orchestrate cytoskeletal dynamics may serve as upstream regulators of mitocytosis. Among these enzymes, polo‐like kinase 1 (PLK1), a serine/threonine kinase known for its role in cell cycle regulation [26] and cytoskeletal organization [27, 28], directly phosphorylates tubulin and microtubule‐associated proteins to regulate microtubule stability [29, 30]. Critically, PLK1 colocalizes with mitochondria and preserves mitochondrial homeostasis, whereas PLK1 deficiency leads to mitochondrial fragmentation, membrane potential loss, and ATP depletion [31, 32]. These findings suggest a potential mechanistic link between PLK1‐mediated cytoskeletal regulation and MQC. However, whether PLK1 directly modulates migrasome‐mediated mitocytosis to sustain efferocytosis after SCI remains unknown.

In this context, we hypothesized that the targeted overexpression of PLK1 in SCI‐resident macrophages promotes mitocytosis, alleviates efferocytosis dysfunction, and mitigates neuroinflammation. To address the challenge of PLK1 delivery, we generated a complex comprising a PLK1‐expressing plasmid and the ROS‐responsive cationic polymer CBP5, which was designed in previous work to achieve efficient gene transfection while limiting cargo‐associated side effects [33]. This biomimetic membrane‐coating strategy enhances biocompatibility and promotes macrophage‐associated delivery within inflammatory SCI lesions, while the ROS‐responsive core provides a subsequent intracellular trigger for plasmid release. In conclusion, this study links PLK1 to mitocytosis and efferocytosis and introduces a macrophage membrane‐coated nanodelivery strategy that provides new insights into functional recovery after SCI (Figure 1).

**FIGURE 1:**
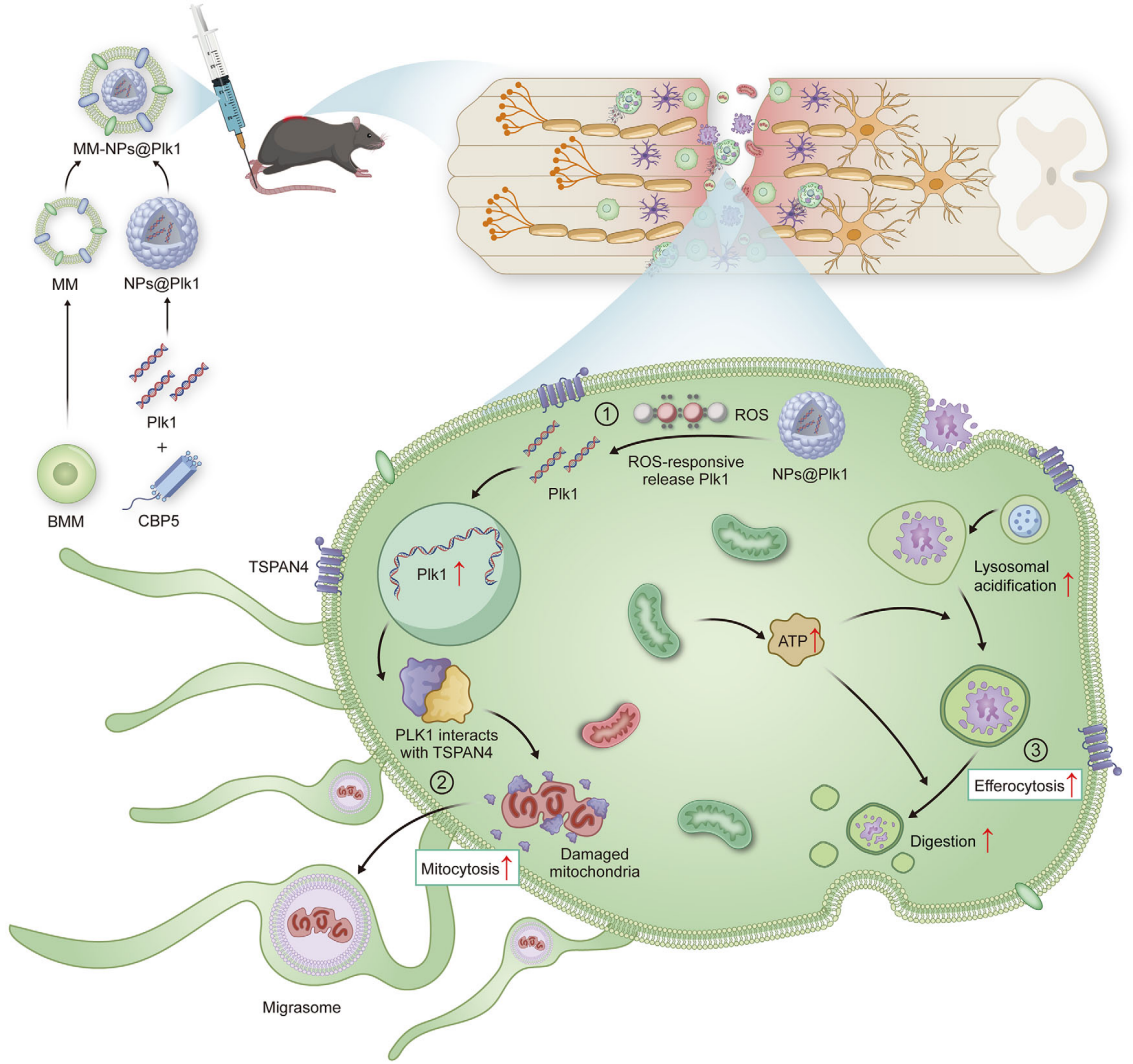
Schematic Illustration of the Therapeutic Mechanism of MM‐NPs@PLK1. MM‐NPs@PLK1 are formed by encapsulating PLK1 plasmids within CBP5 and coating the nanocomplexes with macrophage membranes. After macrophage‐associated uptake, the NPs@PLK1 enter the cytoplasm and release the PLK1‐encoding plasmid in response to elevated intracellular ROS levels. PLK1 overexpression facilitates the clearance of damaged mitochondria and maintains mitochondrial homeostasis by regulating TSPAN4‐mediated migrasome formation, ultimately promoting macrophage efferocytosis and tissue repair.

## 2 Results

### 2.1 Single‐Cell RNA Sequencing Data Reveal an Association Between PLK1 Expression and Efferocytosis Activity After Injury

To investigate the pathological changes that occur after SCI, we analysed single‐cell RNA sequencing (scRNA‐seq) data (Figshare17702045) from uninjured and injured spinal cords at different time points. Uniform manifold approximation and projection (UMAP) analysis was used to identify the main cell populations on the basis of typical gene expression patterns (Figure 2A). Comparative analysis revealed substantial shifts in cellular composition following SCI: the size of the neuronal and astrocytic populations markedly decreased, whereas myeloid cells (macrophages/monocytes) began to accumulate by day 3 and reached peak abundance on day 7 (Figure 2B). Gene Ontology (GO) analysis of the differentially expressed genes (DEGs) identified on day 7 post‐injury revealed enrichment in pathways primarily associated with phagocytosis, engulfment, and the regulation of actin cytoskeleton organization (Figure 2C). To characterize the pathological environment in which macrophage efferocytosis occurs after SCI, we assessed the apoptotic cell burden, macrophage lipid loading, tissue energy status, and inflammatory activation. TUNEL staining revealed a progressive accumulation of ACs at the lesion site from day 3 to day 7 after SCI (Figure 2D), indicating an increasing burden of cellular debris requiring clearance. Moreover, MAC2‐positive macrophages exhibited abundant lipid droplets and a foam‐cell‐like morphology (Figure 2E), suggesting substantial lipid loading in lesion‐associated macrophages. ATP levels in tissue progressively decreased during the first 7 days after SCI (Figure 2F), indicating an increasingly energy‐compromised injury microenvironment, while the expression of iNOS, IL‐1β, and IL‐6 remained elevated (Figure 2G–I), reflecting persistent inflammatory activation. Together, these findings indicate that macrophages at SCI lesions are exposed to increasing apoptotic cell burden, lipid loading, metabolic stress, and sustained inflammation. Given the metabolic requirements of continual efferocytosis, these observations prompted us to further investigate whether injury‐related stress impairs macrophage efferocytic capacity.

**FIGURE 2:**
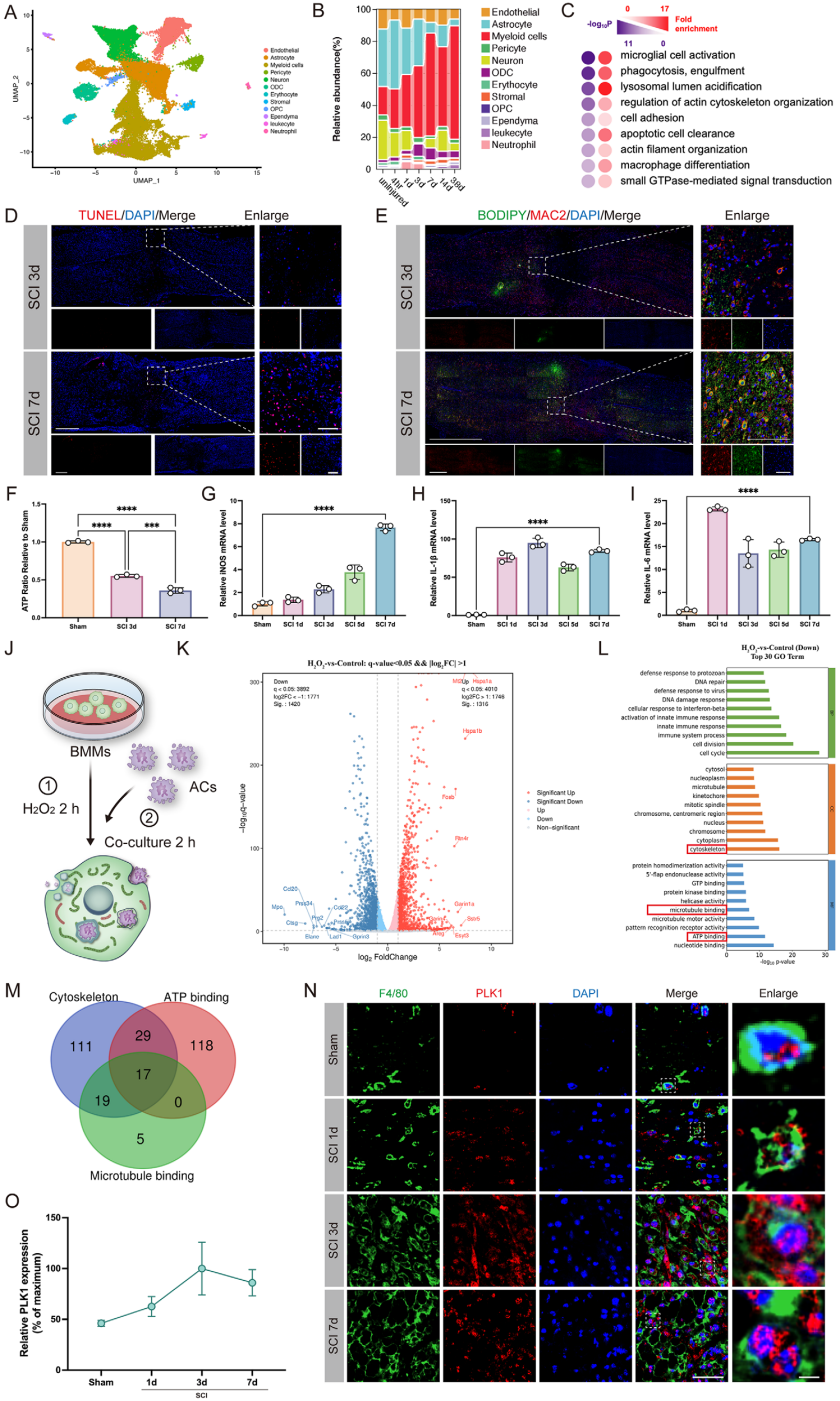
Single‐cell RNA Sequencing Data Reveal an Association between PLK1 Expression and Efferocytosis Activity After Injury. (A) UMAP visualization plot of the results of spinal cord cell sequencing in a database (Figshare17702045). UMAP, uniform manifold approximation and projection. (B) Relative proportions of different cell types in each experimental group. (C) The top 9 pathways from the GO enrichment analysis. GO, Gene Ontology. (D) Representative images of terminal deoxynucleotidyl transferase‐mediated deoxyuridine triphosphate nick end labelling (TUNEL; red) staining of spinal cord sections from SCI mice 3 and 7 days after injury. Scale bar = 500 µm. Enlarged scale bar = 50 µm. (E) The presence of lipid droplets (BODIPY, green) in macrophages (MAC2, red) at 3 and 7 days after SCI. Scale bar = 500 µm. Enlarged scale bar = 50 µm. (F) ATP levels in spinal cord tissues at 3 and 7 days after SCI (n  = 3). (G‐I) RT‒qPCR analysis of the mRNA levels of the inflammatory factors iNOS, IL‐1β, and IL‐6 in spinal cord tissues (n  = 3). (J) Schematic showing the efferocytosis process in vitro. (K) Volcano plot depicting the distribution of DEGs between H_2_O_2_ and control groups. Upregulated genes are marked in red, and downregulated genes are marked in blue. DEGs, differentially expressed genes. (L) The GO enrichment analysis of downregulated DEGs between H_2_O_2_ and control groups shows the top 30 enriched terms in the BP, CC, and MF categories. BP, biological process; CC, cell component; MF, molecular function. (M) Venn diagram comparing downregulated signalling pathways after injury for each dataset. (N) Representative dual immunofluorescence images of spinal cord sections from the indicated groups showing F4/80 (green), PLK1 (red), and nuclei (DAPI, blue) (n = 3). Enlarged images correspond to the boxed regions in the merged images. (O) Quantification of the relative PLK1 fluorescence intensity in F4/80‐positive cells. The data are presented as the mean ± SD. ^***^ *p* <0.001, ^****^ *p* <0.0001.

To further explore the underlying mechanisms, bone marrow‐derived macrophages (BMMs) were treated with H_2_O_2_ or control medium and co‐cultured with ACs to mimic efferocytosis in vitro, after which RNA‐seq analysis was performed (Figure 2J). A comprehensive examination of the gene expression profiles revealed distinct changes in the H_2_O_2_ group, with 1,316 upregulated genes and 1,420 downregulated DEGs compared with those in the control group (Figure 2K). GO enrichment analysis of these DEGs revealed that the downregulated genes were primarily associated with the cytoskeleton in the cellular component category and with microtubule binding and ATP binding in the molecular function category, both of which are processes essential for efferocytosis function (Figure 2L). PLK1 was identified as a potential key regulator on the basis of the enrichment of terms related to the cytoskeleton, microtubule binding, and ATP binding (Figure 2M). Single‐cell RNA‐sequencing analysis revealed that PLK1 transcription in macrophages followed a dynamic temporal pattern after SCI. The relative PLK1 expression level increased modestly on day 1, reached its maximum on day 3, and subsequently decreased on day 7 but remained above the level in the uninjured model (Figure S1). Dual immunofluorescence staining further revealed a PLK1 signal in F4/80‐positive cells. Quantification of PLK1 fluorescence within these cells revealed a similar postinjury temporal pattern (Figures 2N,O). These results identify PLK1 as a candidate regulator of macrophage efferocytosis following SCI.

### 2.2 PLK1 Promotes Macrophage Efferocytosis

To investigate the role of PLK1 in macrophage efferocytosis, PLK1 was first successfully overexpressed in BMMs (Figure S2A–C). PLK1‐overexpressing BMMs were then stimulated with H_2_O_2_ and cocultured with ACs. Immunofluorescence staining revealed that PLK1 markedly promoted macrophage efferocytosis (Figure 3A,B), which was further confirmed by flow cytometry (Figure 3C–E). Moreover, an IncuCyte live‐cell imaging system was used to monitor continual efferocytosis in real time. As shown in Figure 3F, the H_2_O_2_ group presented markedly reduced macrophage phagocytic capacity, whereas PLK1 overexpression restored and enhanced this capacity. Five hours after the addition of ACs, the percentage of engulfed ACs began to decrease, indicating that the exposure of macrophages to H_2_O_2_ impaired continual efferocytosis. To further validate this effect, we first incubated the macrophages with green fluorescent ACs for 45 min; then, 2 h after the ACs were removed, the cells were incubated with red fluorescent ACs for 45 min. The proportion of macrophages that internalized both green and red ACs was significantly greater in the PLK1‐overexpressing group than in the injury group, indicating increased capacity for continual efferocytosis (Figure 3G,H). Furthermore, PLK1 overexpression upregulated the expression of anti‐inflammatory factors (IL‐10 and YM‐1) (Figure 3I,J), suggesting that promotion of efferocytosis is accompanied by an increase in anti‐inflammatory signalling.

**FIGURE 3:**
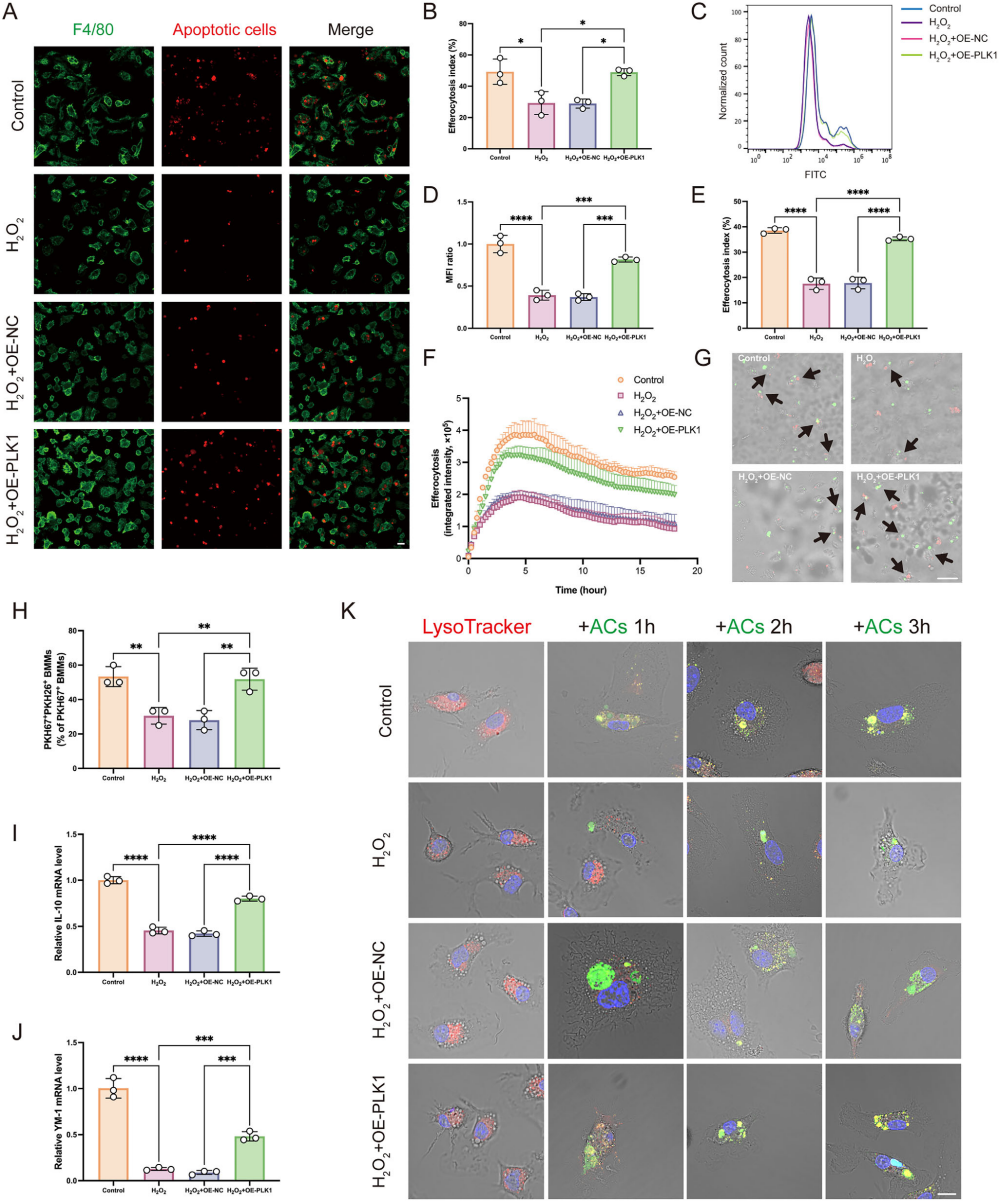
PLK1 Promotes Macrophage Efferocytosis. (A and B) Dual immunofluorescence staining (A) and quantification (B) of BMMs (F4/80, green) and ACs (PKH26, red) (n = 3). Scale bar = 50 µm. BMMs, bone marrow–derived macrophages. ACs, apoptotic cells. (C) AC‐positive BMMs determined by flow cytometry after treatment (n = 3). (D) MFI of CFSE (n = 3). MFI, mean fluorescence intensity. CFSE, carboxyfluorescein succinimidyl ester. (E) Quantitative analysis of efferocytosis (n = 3). (F) The intensity of red objects (i.e., internalized ACs) expressed as RCU × µm^2^/image by Incucyte at different time points after the addition of pHrodo red–labelled ACs to BMMs. RCU, red calibrated unit. (G and H) BMMs were incubated with PKH67‐labelled ACs (green) for 45 min, followed by washing and a 2‐h rest period prior to incubation with PKH26‐labelled ACs (red) for 45 min, and the number of PKH67^+^PKH26^+^ BMMs (green and red; black arrows) was quantified (H) relative to that of PKH67^+^ BMMs (green only), which reflects continual efferocytosis (n  = 3). (I and J) RT‒qPCR analysis of the mRNA levels of the inflammatory factors IL‐10 and YM‐1 in BMMs (n  = 3). (K) Representative confocal image showing phagosome acidification in BMMs after efferocytosis. BMMs were cocultured with CFSE‐labelled ACs (green) for 1, 2, or 3 h. The nuclei were stained with Hoechst (blue), and phagosome acidification was indicated by LysoTracker Deep Red (red). Scale bars = 10 µm. The data are presented as the mean ± SD. ^*^ *p* <0.05, ^**^ *p* <0.01, ^***^ *p* <0.001, ^****^ *p* <0.0001.

We next examined the specific stage of the efferocytosis process affected by PLK1. Our previous in vivo observations revealed that macrophages in SCI lesions often contain numerous lipid droplets and apoptotic fragments, suggesting that the recognition and engulfment phases of efferocytosis are largely preserved after SCI (Figure 2D,E). Notably, Raymond et al. [34] demonstrated that the digestion phase is the rate‐limiting step of efferocytosis and that macrophages overloaded with undigested ACs exhibit markedly reduced capacity for continual clearance. These findings indicate that digestion defects, rather than recognition or phagocytosis failures, underlie efferocytosis dysfunction following SCI. Given that efficient efferocytosis ultimately depends on the digestion of apoptotic cargo within phagolysosomes, we next assessed phagolysosomal acidification using the pH‐sensitive dye LysoTracker. We found that PLK1 significantly promoted phagolysosome formation and acidification within three hours (Figure 3K), indicating that PLK1 is essential for the maturation of phagolysosomes and the digestion of apoptotic cargo.

### 2.3 PLK1 Regulates Mitochondrial Function

Efferocytosis requires a large amount of energy, which is provided primarily by mitochondria. Previous studies have shown that improving mitochondrial function can promote efferocytosis, thereby reducing inflammatory responses [35]. Therefore, we investigated whether PLK1 modulates mitochondrial function in macrophages. GO enrichment analysis of DEGs between the H_2_O_2_+NC and H_2_O_2_+PLK1 groups revealed the upregulation of genes related to actin cytoskeleton organization and cell migration and the downregulation of genes associated with inflammatory and innate immune responses in the H_2_O_2_+PLK1 group (Figure 4A). Gene set enrichment analysis (GSEA) further revealed that genes involved in the actin cytoskeleton were enriched in the H_2_O_2_+PLK1 group, while apoptotic pathways were suppressed (Figure 4B). Given that both the engulfment and digestion phases of efferocytosis rely on actin rearrangements, which in turn demand a substantial energy supply [36], we further explored the potential link between PLK1 and mitochondria. Using confocal microscopy, we observed that PLK1 colocalized with the mitochondrial marker TOM20 (Figure 4C), suggesting an association between PLK1 and mitochondria, which is consistent with prior reports [32]. Functionally, PLK1 overexpression significantly decreased mitochondrial ROS levels according to the MitoSOX analysis (Figure 4D,E). Additionally, we evaluated the MMP, a sensitive indicator of mitochondrial function. The MMP was markedly reduced in the H_2_O_2_ group, whereas PLK1 promoted its recovery (Figure 4F). Quantitative analysis further revealed increased red fluorescence intensity and a significant decrease in JC‐1 monomers in the PLK1 overexpression group (Figure 4G), supporting the conclusion that PLK1 helps restore mitochondrial function. In addition, we used IncuCyte real‐time live‐cell imaging combined with tetramethylrhodamine methyl ester (TMRM), a membrane potential‐sensitive fluorescent probe, to monitor mitochondrial membrane potential dynamics. The TMRM signal, expressed as red calibrated units (RCU), reflects the overall mitochondrial membrane potential level of the macrophages. Our results revealed that the TMRM RCU value was markedly reduced in the H_2_O_2_ group and the H_2_O_2_+NC group, indicating that oxidative stress induced mitochondrial depolarization, whereas this value was significantly higher in the PLK1 overexpression group, suggesting that mitochondrial membrane potential and mitochondrial homeostasis were restored at the cellular level (Figure 4H). Moreover, PLK1 overexpression increased ATP production (Figure 4I). Together, these findings demonstrated that PLK1 overexpression alleviates mitochondrial dysfunction and sustains the metabolic demands of efferocytosis.

**FIGURE 4:**
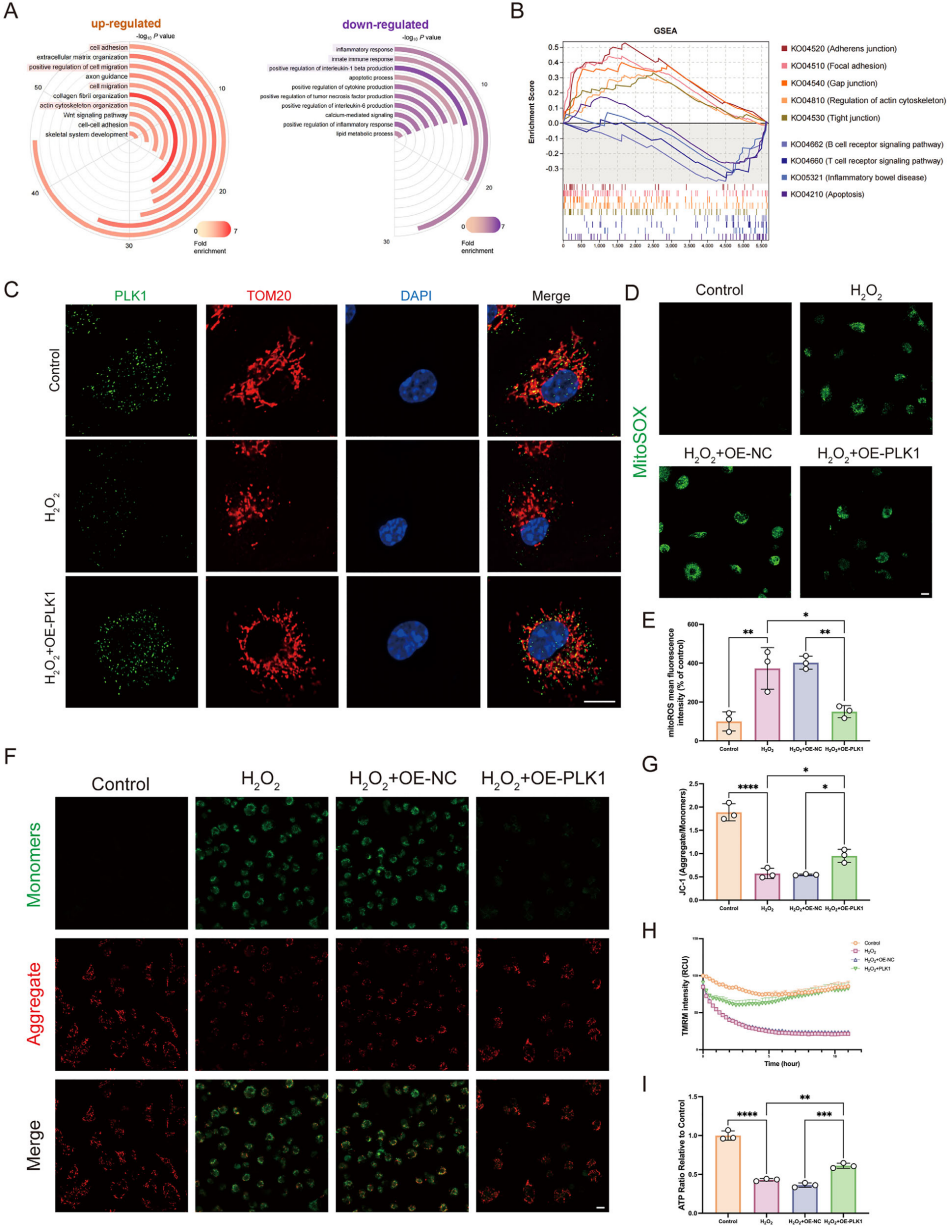
PLK1 Regulates Mitochondrial Function. (A) Top 10 pathways from the GO enrichment analysis between H_2_O_2_‐NC and H_2_O_2_‐PLK1. (B) GSEA of DEGs between H_2_O_2_‐NC and H_2_O_2_‐PLK1. GSEA, gene set enrichment analysis. (C) Dual immunofluorescence staining of PLK1 (green) and TOM20 (red). Scale bar = 20 µm. (D and E) Analysis of mitochondrial ROS levels. The green fluorescence intensity of MitoSOX represents the mitochondrial ROS level (n  = 3). Scale bar = 10 µm. (F and G) Analysis of the mitochondrial membrane potential (MMP). The ratio of red to green fluorescence intensity in JC‐1 staining (n  = 3). Scale bar = 10 µm. (H) The intensity of tetramethylrhodamine methyl ester (TMRM) of the MMP expressed as RCU × µm^2^/image by IncuCyte at different time points in each group (n  = 3). (I) ATP levels in BMMs (n  = 3). The data are presented as the mean ± SD. ^*^ *p* <0.05, ^**^ *p* <0.01, ^***^ *p* <0.001, ^****^ *p* <0.0001.

### 2.4 PLK1 Promotes Mitocytosis by Regulating TSPAN4‐Mediated Migrasomes

Given that our previous GO enrichment analysis revealed enrichment of cell migration‐related genes in the PLK1 overexpression group (Figure 4A) and that migrasome‐mediated mitocytosis has recently emerged as an important mechanism for maintaining mitochondrial function and restoring normal cellular phenotypes under injury‐related stress [21, 24, 37], we further investigated whether PLK1 influences this process in macrophages. First, we visualized the migrasomes by staining them with wheat germ agglutinin (WGA‐FITC, green fluorescence). The results revealed a marked increase in the number of migrasomes in the PLK1 overexpression group compared with that in the H_2_O_2_‐treated group (Figure 5A). Furthermore, compared with H_2_O_2_ treatment, PLK1 overexpression significantly increased the number of mitochondria within the migrasomes (Figure 5B,C), suggesting that PLK1 promotes the removal of damaged mitochondria via migrasomes. Since TSPAN4 is a key regulator of migrasome formation, we next validated the potential interaction between PLK1 and TSPAN4 using molecular docking analysis. The results indicated strong binding affinity between PLK1 and TSPAN4, with a docking score of −289.2 (Figure 5D). We further performed reciprocal coimmunoprecipitation (co‐IP) experiments to examine the association between PLK1 and TSPAN4. Immunoprecipitation of PLK1 followed by immunoblotting for TSPAN4 demonstrated that TSPAN4 coimmunoprecipitated with PLK1. Conversely, immunoprecipitation of TSPAN4 followed by immunoblotting for PLK1 demonstrated that PLK1 coimmunoprecipitated with TSPAN4 (Figure 5E). No corresponding signals were detected in the IgG controls. Together, these reciprocal co‐IP results support an association between PLK1 and TSPAN4 in BMMs.

**FIGURE 5:**
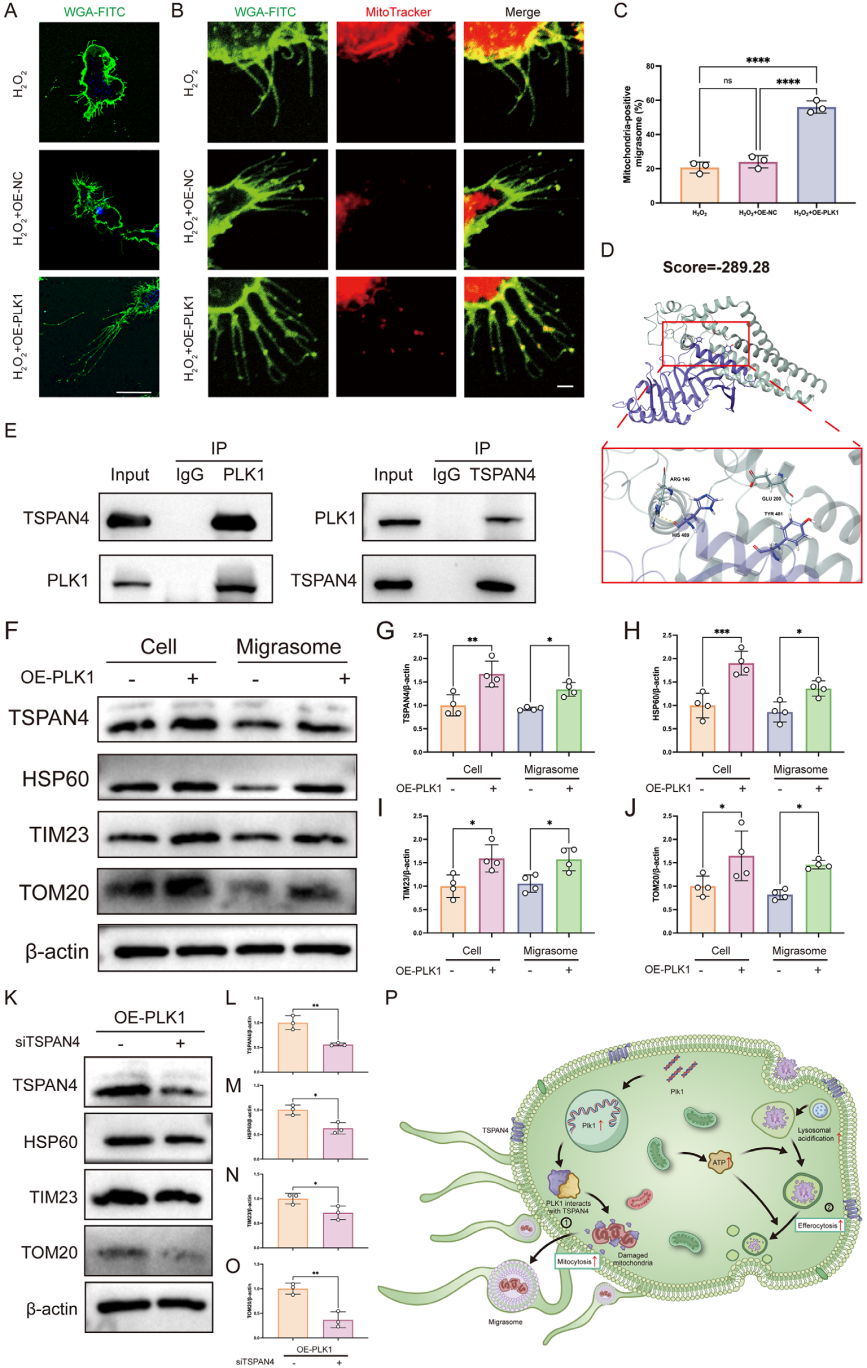
PLK1 Promotes Mitocytosis by Regulating TSPAN4‐mediated Migrasomes. (A) Immunofluorescence staining of migrasomes (WGA‐FITC, green) (n  = 3). WGA, wheat germ agglutinin. Scale bar = 20 µm. (B, C) Dual immunofluorescence staining (B) and quantification (C) of migrasomes (WGA‐FITC, green) and mitochondria (MitoTracker, red) (n  = 3). Scale bar = 20 µm. (D) Molecular docking analysis of PLK1 and TSPAN4. (E) Co‐IP analysis of the interaction between PLK1 and TSPAN4 in BMMs was performed with anti‐PLK1 or anti‐TSPAN4 primary antibodies. (F–J) Western blot analysis and quantification of the expression of the migrasome marker and mitochondrial‐related proteins in the indicated BMMs and migrasomes isolated from BMMs (n  =  4). (K–O) Western blot analysis and quantification of the expression of the migrasome marker and mitochondrial‐related proteins in the indicated migrasomes isolated from BMMs with PLK1 overexpression and TSPAN4 knockdown (n = 3). (P) Schematic illustration of the mechanism by which PLK1 regulates migrasome‐mediated mitocytosis. The data are presented as the mean ± SD. ^*^ *p* <0.05, ^**^ *p* <0.01, ^***^ *p* <0.001, ^****^ *p* <0.0001.

Next, we extracted migrasomes from BMMs with or without PLK1 treatment. Western blot analysis revealed elevated expression of the migrasome marker TSPAN4, as well as mitochondrial proteins—including TOM20 (outer membrane), TIM23 (inner membrane), and HSP60 (matrix)—in both PLK1‐overexpressing cells and isolated migrasomes (Figure 5F–J), indicating that PLK1 enhances migrasome formation and promotes the enrichment of mitochondrial proteins within migrasomes. To further assess the functional role of TSPAN4 in PLK1‐driven mitocytosis, we knocked down or overexpressed TSPAN4 in PLK1‐treated BMMs. Western blot analysis demonstrated that TSPAN4 knockdown reduced the expression of TSPAN4, TOM20, TIM23, and HSP60 (Figure 5K–O), whereas TSPAN4 overexpression had the opposite effect (Figure S3A–E). We also performed experiments with PLK1 knockdown and TSPAN4 overexpression in BMMs. Western blot analysis of isolated migrasomes revealed that PLK1 knockdown decreased the levels of TSPAN4 and the mitochondrial proteins TOM20, TIM23, and HSP60. Importantly, TSPAN4 overexpression partially reversed these changes induced by PLK1 knockdown (Figure S3F–J). Consistent with these findings, WGA/MitoTracker fluorescence imaging revealed reduced mitochondrial incorporation into migrasomes following PLK1 knockdown, which was partially restored by TSPAN4 overexpression (Figure S3K). Together, these reciprocal rescue experiments support a role for TSPAN4 downstream of PLK1 in regulating migrasome‐mediated mitocytosis. In summary, these findings indicated that PLK1 facilitates the clearance of damaged mitochondria through TSPAN4‐dependent migrasome formation, thereby sustaining mitochondrial quality and macrophage efferocytic capacity (Figure 5P).

### 2.5 Fabrication and Characterization of MM‐NPs@PLK1

We previously demonstrated that PLK1 clears damaged mitochondria by facilitating mitocytosis, thereby increasing macrophage efferocytic capacity. Given the increase in ROS levels in macrophages associated with injury [38], we engineered a complex of our previously designed ROS‐responsive cationic polymer CBP5 and PLK1‐expressing plasmids (NPs); then, we extracted the cell membranes of BMMs (MMs) to package the NPs to enable their effective delivery within the post‐injury microenvironment (Figure 6A). RAW264.7 cells transfected with luciferase plasmids were encapsulated using NPs at varying N/P ratios and cultured in medium supplemented with either 0% or 10% foetal bovine serum (FBS). NPs with an N/P ratio of 20 demonstrated optimal transfection efficiency in medium without FBS (Figure 6B). At the optimal N/P ratio of 20, the cationic polymer NPs efficiently condensed DNA into homogeneous and stable nanocomplexes, with an average diameter of approximately 80.4 nm and a zeta potential of 14.4 mV (Figure 6C and Figure S4A). Following membrane coating, the size and zeta potential of the MM‐NPs were similar to those of the MM. The resulting MM‐NPs significantly increased in size to 132.0 nm, while their zeta potential (−24.6 mV) was close to that of the MM vesicles (−24.3 mV) (Figure 6D–G). This charge reversal, together with the increase in size, confirmed successful cell membrane encapsulation. Additionally, the role of ROS in modulating NP‐mediated transfection was assessed by analysing luciferase activity after different H_2_O_2_ treatments (Figure S4B). Gel electrophoresis revealed that H_2_O_2_ markedly increased DNA release from the NPs@DNA, verifying their ability to release ROS in a ROS‐responsive manner (Figure 6H).

**FIGURE 6:**
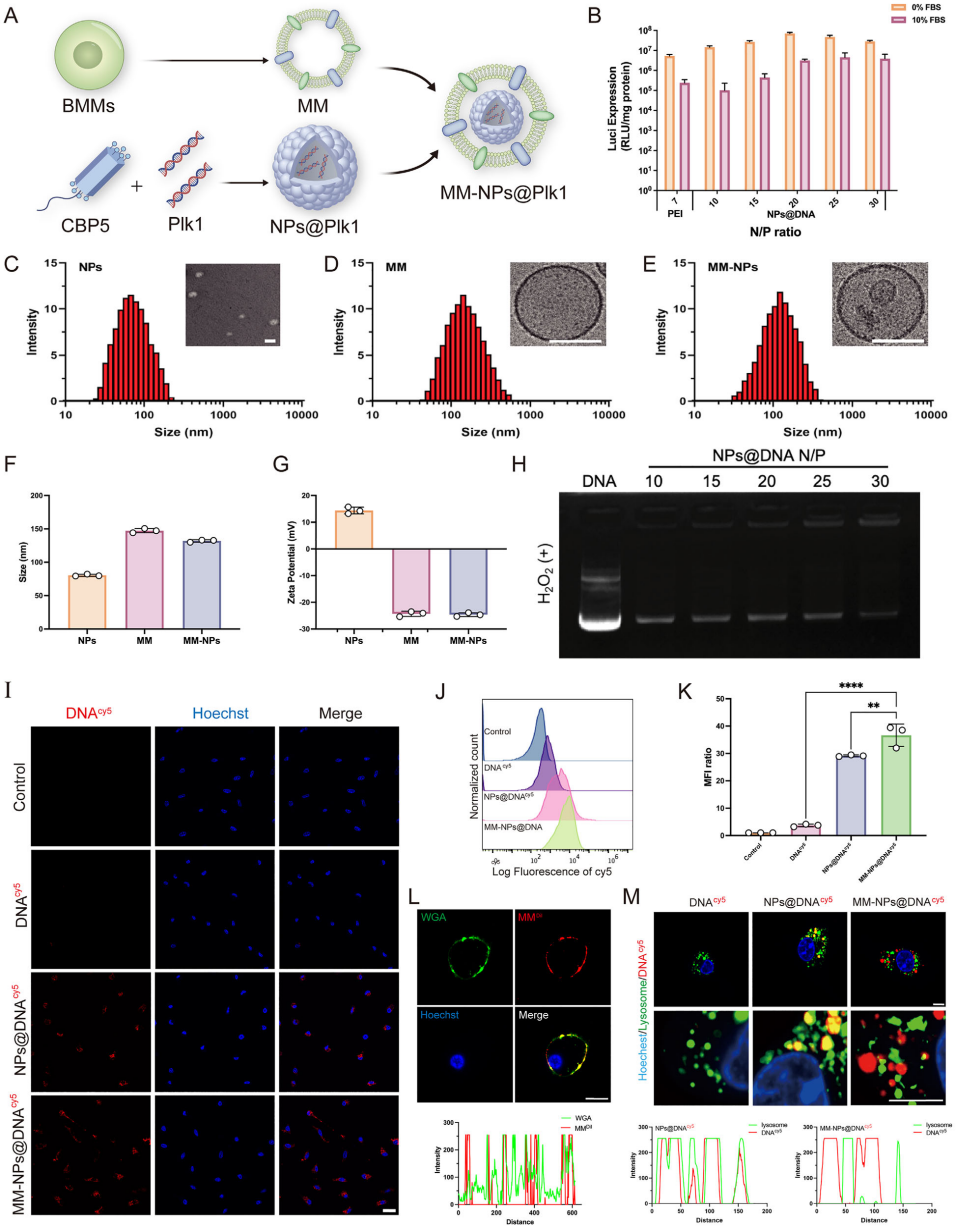
Fabrication and Characterization of MM‐NPs@PLK1. (A) Schematic of plasmid PLK1 loading into CBP5 (NPs@PLK1) encapsulated by a macrophage membrane (MM‐NPs@PLK1). (B) Luciferase expression in RAW264.7 cells treated with NPs@DNA at different N/P ratios with or without 10% serum‐containing medium (n = 3). (C) Size and TEM image of the NPs. Scale bar = 100 nm. TEM, transmission electron microscopy. (D) Size and cryo–electron microscopy image of the MM. Scale bar = 100 nm. (E) Size and cryo–electron microscopy image of the MM‐NPs. Scale bar = 100 nm. (F) Mean diameters of the NPs, MM, and MM‐NPs. (G) The surface zeta potential of the NPs, MM, and MM‐NPs. (H) Gel retardation assay of NPs@DNA at different N/P ratios after a 1‐h incubation at 37°C with H_2_O_2_. (I) Representative fluorescence visualization showing the uptake of DNA by BMMs. DNA, red; Hoechst, blue. Scale bars = 20 µm. (J) Cy5‐positive BMMs were measured by flow cytometry after treatment with DNA^Cy5^, NPs@DNA^Cy5^, or MM‐NPs@DNA^Cy5^ (n  =  3). (K) MFI of Cy5 (n  =  3). (L) Immunofluorescence staining and intensity of WGA‐labelled BMMs (green) and DiI‐labelled MM (red). Scale bar = 20 µm. (M) Fluorescence visualization of DNA^Cy5^ and lysosome localization in BMMs after incubation with DNA^Cy5^, NPs@DNA^Cy5^, and MM‐NPs@DNA^Cy5^ for 2 h, and intensity profiles across the cell. DNA, red; lysosomes, green; nuclei, blue. Scale bar = 10 µm. Enlarged scale bars = 5 µm. The data are presented as the mean ± SD. ^**^ *p* <0.01, ^****^ *p* <0.0001.

To elucidate the function of MM‐NPs@PLK1, we first investigated the interaction between MM‐NPs@DNA^Cy5^ and BMMs. As shown in Figure 6I, ^DNA Cy5^ was efficiently internalized by the BMMs after 4 h of incubation. Moreover, more MM‐NPs@DNA^Cy5^ than NPs@DNA^Cy5^ were internalized in medium containing 10% serum. These results indicated that MM increases the serum stability of the NPs and promotes their internalization by BMMs (Figure 6I–K). We then studied the intracellular trafficking properties of the MM‐NPs. MM^DiI^‐NPs (red) were incubated with BMMs (labelled with WGA, green) for 1 h. Most of the red and green fluorescence overlapped, and yellow puncta were observed by confocal microscopy (Figure 6L). On the basis of the observed colocalization between MM‐NPs and their homologous membranes, we investigated the potential pathways through which MM‐NPs are taken up by cells. Flow cytometry analysis revealed that cellular uptake of the MM‐NPs was suppressed at 4°C but not by endocytosis inhibitors (chlorpromazine, wortmannin, filipin III, or cytochalasin D) (Figure S4C,D). These data suggest that MM‐NP entry occurs primarily through membrane fusion rather than through classical endocytic mechanisms.

Further analysis using LysoTracker Green‐labelled BMMs revealed that compared with treatment with free DNA^Cy5^, treatment with MM‐NPs@DNA^Cy5^ and NPs@DNA^Cy5^ increased Cy5 fluorescence intensity. Notably, the localization of MM‐NPs@DNA^Cy5^ in lysosomes significantly decreased after 2 h of incubation (Figure 6M), indicating efficient lysosomal escape. Collectively, these results demonstrate a membrane fusion‐mediated delivery mechanism for MM‐NPs that bypasses lysosomal degradation, establishing a strategic platform for enhanced therapeutic delivery of DNA.

### 2.6 In Vivo Targeting Ability of MM‐NPs@PLK1

Following the confirmation of efficient MM‐NPs@PLK1 delivery in vitro, we further evaluated the in vivo targeting ability and biosafety of these nanoparticles in a mouse model of SCI. To assess the targeting ability of MM‐NPs@PLK1, mice were administered free indocyanine green (ICG) or ICG‐loaded macrophage membrane‐coated nanoparticles (MM@ICG). In vivo fluorescence imaging was performed to evaluate the accumulation of MM@ICG in the spinal cord region of live SCI mice. MM@ICG produced a detectable fluorescence signal at the injured spinal cord region as early as 6 h after administration, with sustained retention observed for up to 72 h (Figure 7A). In contrast, the fluorescence signal of free ICG in the lesion region was markedly weaker and declined more rapidly. Quantitative analysis of the fluorescence intensity within a fixed region of interest centred on the lesion confirmed the significantly greater accumulation of MM@ICG than of free ICG (Figure 7B). Together, these results demonstrate the enhanced and prolonged accumulation of MM@ICG in the injured spinal cord in vivo.

**FIGURE 7:**
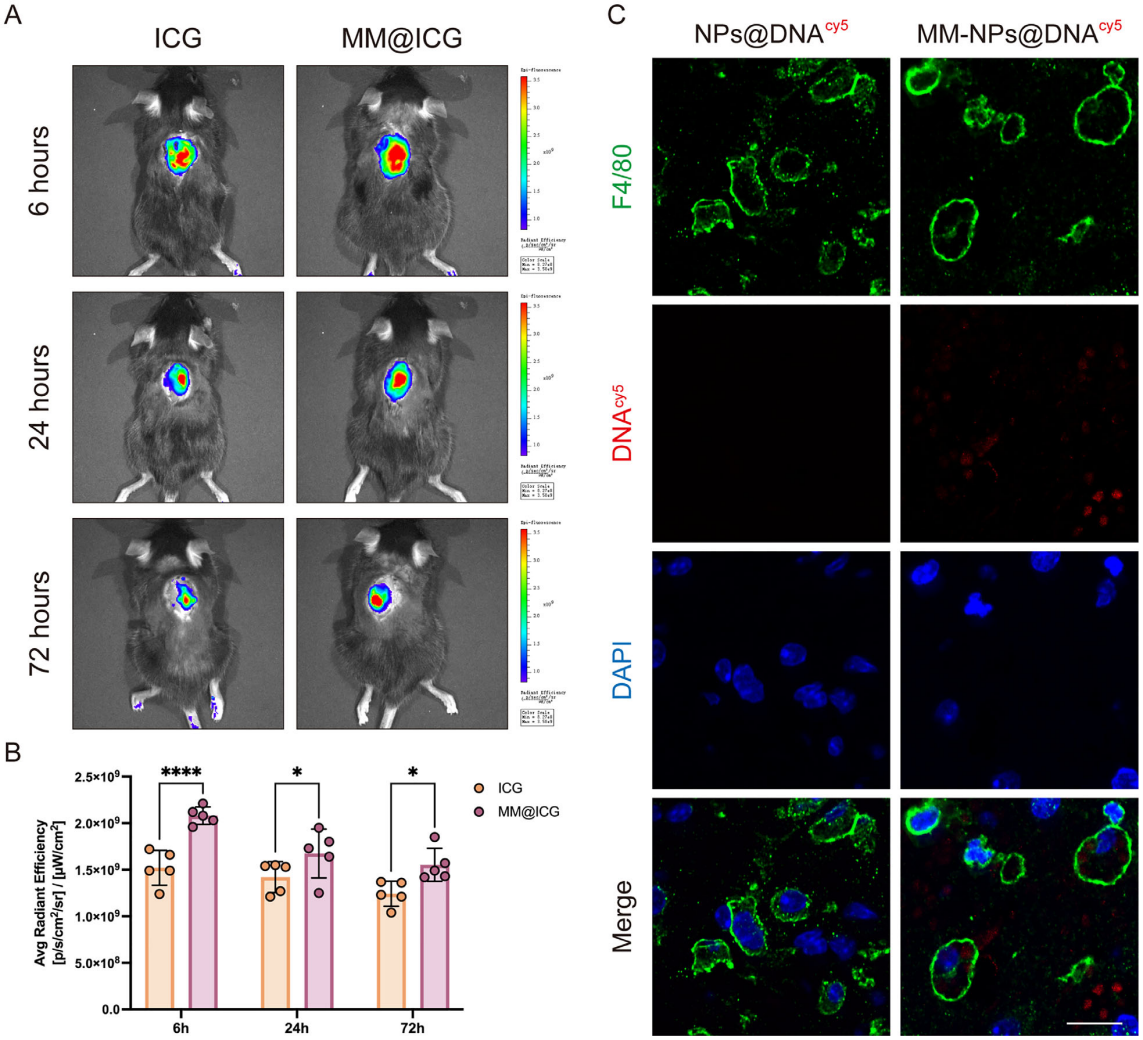
In Vivo Targeting Ability of MM‐NPs@PLK1. (A) Representative in vivo fluorescence images of SCI mice acquired using an IVIS system at 6, 24, and 72 h after intravenous administration of free ICG or MM@ICG (n = 5). (B) Quantitative analysis of radiant efficiency within a fixed region of interest centred on the injured spinal cord in (A). (C) Fluorescence images of spinal cord sections stained with an anti‐F4/80 antibody (green) after treatment with NPs@DNA^Cy5^ or MM‐NPs@DNA^Cy5^ (red). Scale bars = 10 µm. The data are presented as the mean ± SD. ^*^ *p* <0.05, ^****^ *p* <0.0001.

To evaluate the cellular distribution of MM‐NPs at the SCI lesion, Cy5‐labelled DNA was used to track the nanocomplexes, and spinal cord sections were stained with the macrophage‐associated marker F4/80. Distinct DNA^Cy5^ fluorescent puncta were observed within F4/80‐positive cells after MM‐NPs@DNA^Cy5^ administration, supporting the idea that MM‐NPs are associated with and taken up by F4/80‐positive macrophages at the injury site (Figure 7C). Together with the enhanced uptake of MM‐NPs@DNA^Cy5^ by BMMs observed in vitro, these results indicate that the macrophage membrane coating promotes macrophage‐associated delivery. In addition, we evaluated the biosafety of MM‐NPs@PLK1 by performing haematoxylin and eosin (H&E) staining on major organ tissues (heart, liver, spleen, lung, and kidney). No significant pathological abnormalities were detected (Figure S5A). Moreover, quantitative haematological and biochemical analyses, including WBC (white blood cells), RBC (red blood cells), HGB (haemoglobin), PLT (platelets), AST (aspartate aminotransferase), ALT (alanine aminotransferase), BUN (blood urea nitrogen‌), and UA (uric acid‌) analyses, were performed. The results revealed no significant difference among these groups (Figure S5B). In summary, combined histological and biochemical blood analyses demonstrated that MM‐NPs@PLK1 have a favorable biosafety profile.

### 2.7 MM‐NPs@PLK1 Promote Mitocytosis to Increase Efferocytosis After SCI

To further investigate the in vivo protective and therapeutic effects of MM‐NPs@PLK1 in SCI, we treated SCI model mice with saline, MM, MM‐NPs@NC, or MM‐NPs@PLK1. We first assessed the effects of the nanoplatform on migrasome formation and mitocytosis. Western blot analysis revealed that compared with the SCI, MM, and MM‐NPs@NC groups, the MM‐NPs@PLK1 group had markedly increased TSPAN4 protein levels, indicating increased migrasome formation (Figure 8A,B). This was accompanied by elevated levels of the mitochondrial proteins TOM20, TIM23, and HSP60 (Figure 8A–E). In addition, ATP levels in the spinal cord were significantly increased in the MM‐NPs@PLK1 group (Figure 8F). Collectively, these in vivo findings align with our in vitro data, confirming that MM‐NPs@PLK1 sustain mitochondrial mass homeostasis through the promotion of migrasome‐mediated mitocytosis.

**FIGURE 8:**
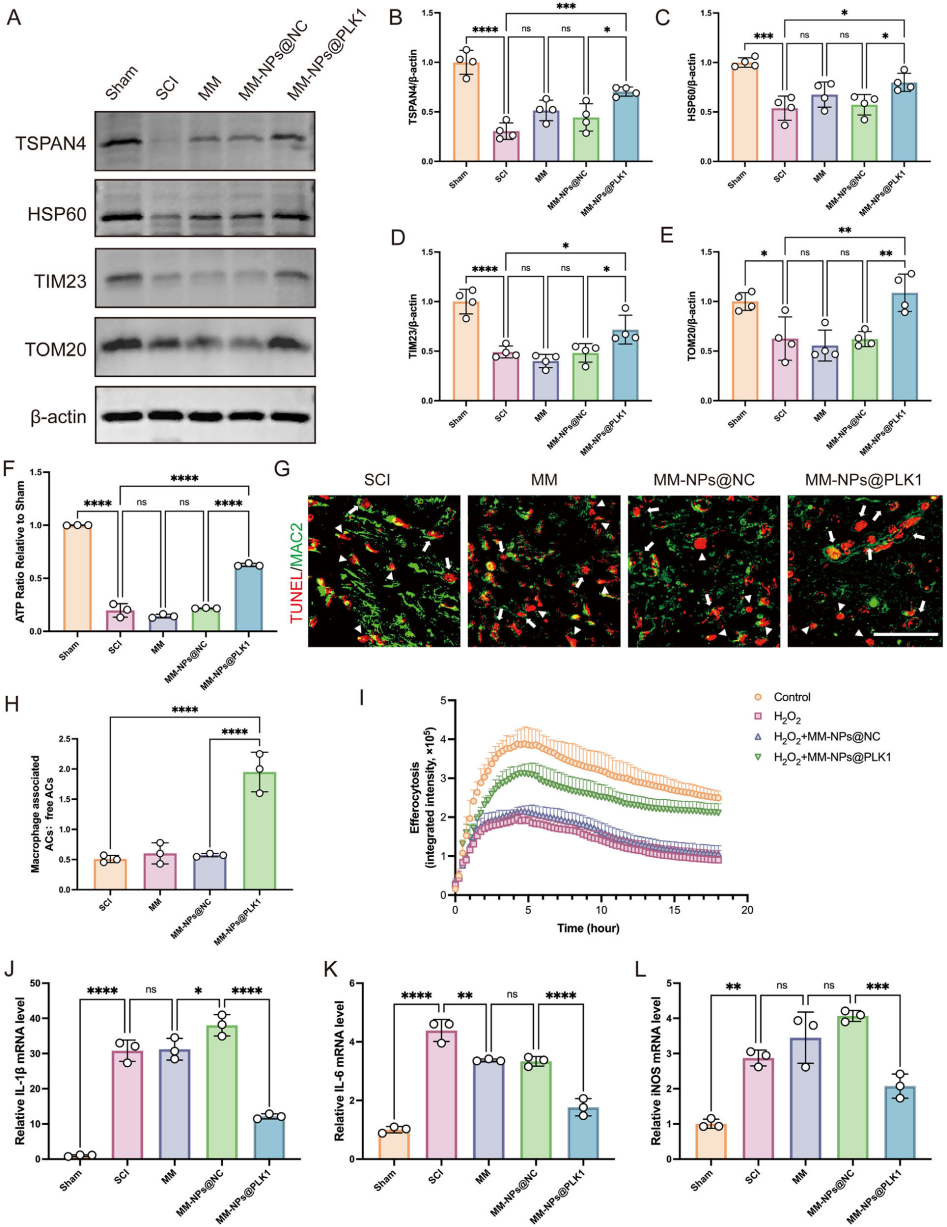
MM‐NPs@PLK1 Promote Mitocytosis to Increase Efferocytosis after SCI. (A–E) Western blot analysis (A) and quantification (B–E) of the expression of migrasome markers and mitochondrion‐related proteins in the indicated spinal cord (n  =  4). (F) ATP levels in spinal cord tissues at 7 days after treatment (n  =  3). (G, H) Dual immunofluorescence staining (G) and quantification (H) of macrophages (MAC2, green) and ACs (TUNEL, red) to evaluate efferocytosis (macrophage‐associated ACs: free ACs) in vivo. Arrowheads and arrows depict macrophage‐associated and free TUNEL^+^ cells, respectively. Scale bar = 50 µm. (I) The intensity of red fluorescence (i.e., internalized ACs) is expressed as RCU × µm^2^/image by IncuCyte at different time points after the addition of pHrodo red–labelled ACs to BMMs after treatment. (J–L) RT‒qPCR analysis of the mRNA levels of the inflammatory factors IL‐1β, IL‐6, and iNOS in the spinal cords of the mice in each group (n = 3). The data are presented as the mean ± SD. ^*^ *p* <0.05, ^**^ *p* <0.01, ^***^ *p* <0.001, ^****^ *p* <0.0001.

We then evaluated whether these in vivo changes were accompanied by improved efferocytosis and inflammation resolution. To evaluate macrophage efferocytic capacity, we quantified the ratio of macrophage‐associated ACs to free ACs in each group using immunofluorescence staining. As shown in Figure 8G,H, the MM‐NPs@PLK1 group exhibited a significantly greater ratio of macrophage‐associated ACs to free ACs. In parallel, IncuCyte live‐cell imaging revealed enhanced macrophage efferocytosis after MM‐NPs@PLK1 treatment in vitro (Figure 8I). In addition, the mRNA expression of the proinflammatory factors IL‐1β, IL‐6, and iNOS was significantly decreased in the MM‐NPs@PLK1 group (Figure 8J–L). Taken together, these data confirmed that MM‐NPs@PLK1 promote macrophage efferocytosis in SCI model mice by promoting migrasome‐mediated mitocytosis, thereby ameliorating the impairment of core mitochondrial dysfunction and efferocytosis that perpetuates secondary SCI.

### 2.8 MM‐NPs@PLK1 Support Neural Structural Preservation and Improve Motor Function Recovery After SCI

Neural structural preservation and circuit repair are important components of functional recovery after SCI [39]. To evaluate the effect of MM‐NPs@PLK1 on neural tissue architecture after SCI, we first analysed the pathological morphology of spinal cord tissues via H&E staining at 28 days post‐SCI. Extensive scar formation and large cavitations were observed near the lesion site in the SCI, MM, and MM‐NPs@NC groups, whereas these pathological features were attenuated by MM‐NPs@PLK1 treatment (Figure 9A). We next performed immunofluorescence staining for NF200 (an axonal marker), MAP2 (a neuronal dendritic marker), and GFAP (an astrocytic marker). In the SCI, MM, and MM‐NPs@NC groups, dense GFAP‐positive astroglial scars were observed around the rostral and caudal margins of the lesion, with limited NF200‐positive axonal structures and MAP2‐positive neural signals extending into the lesion core (Figure 9B). In contrast, MM‐NPs@PLK1 treatment was associated with reduced GFAP‐positive astroglial scarring, a greater abundance of NF200‐positive structures near the lesion margins, and stronger MAP2 immunoreactivity within the lesion region (Figure 9B). These histological features were consistent with improved neural structural preservation after SCI. Western blot analysis further revealed increased NF200 and MAP2 protein levels in the MM‐NPs@PLK1 group (Figure 9C–E). Collectively, these data indicate that MM‐NPs@PLK1 facilitate neural structural preservation after SCI, thereby establishing a structural foundation for functional recovery.

**FIGURE 9:**
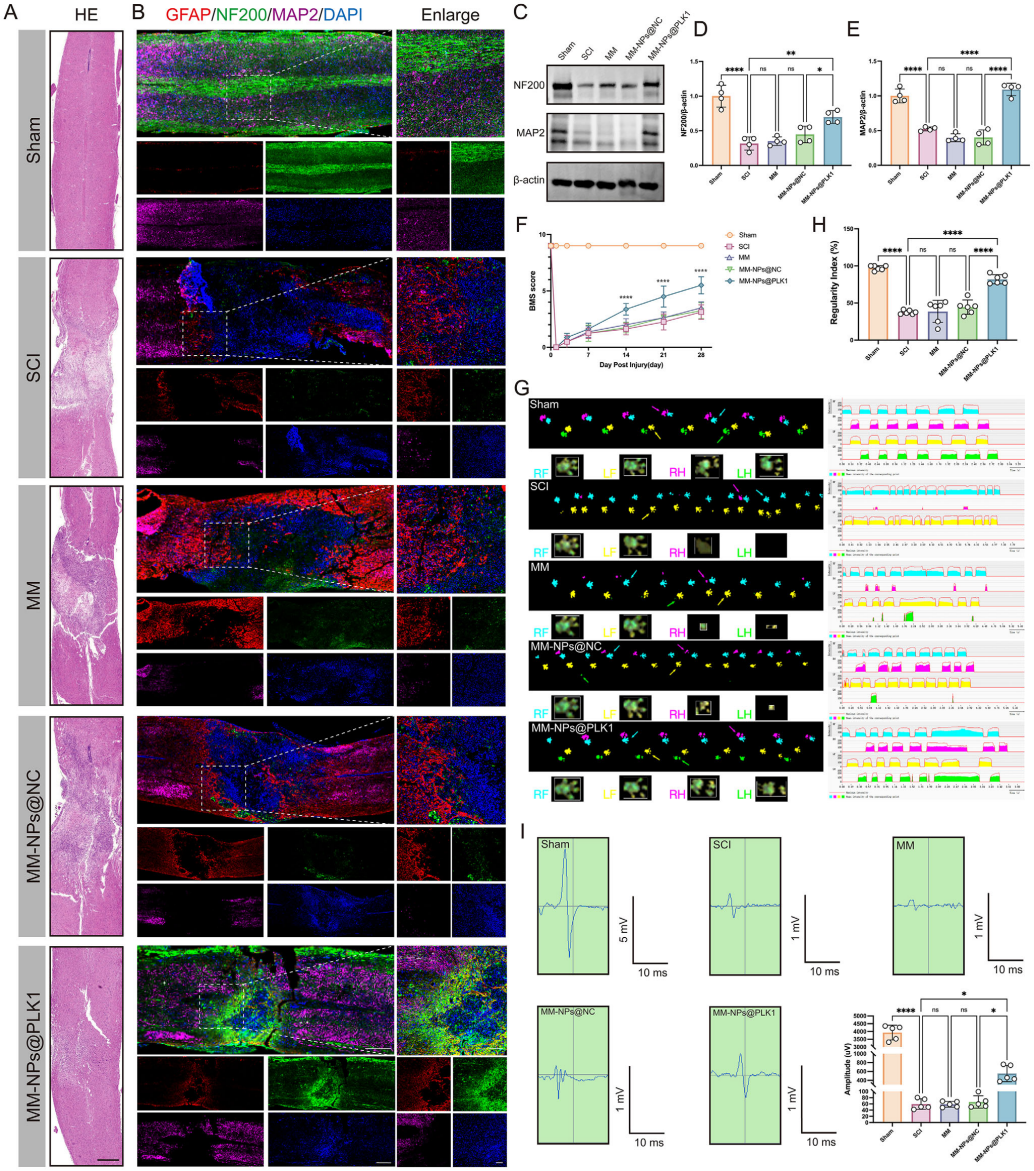
MM‐NPs@PLK1 Support Neural Structural Preservation and Improve Motor Function Recovery after SCI. (A) Representative H&E staining images 28 days after SCI. Scale bar = 500 µm. (B) Representative triple immunofluorescence images of GFAP (red), NF200 (green), MAP2 (purple), and nuclei (DAPI, blue) staining in spinal cord tissues from different groups. Scale bar = 500 µm. Enlarged scale bar = 100 µm. (C‐E) Western blot analysis (C) and quantification (D and E) of NF200 and MAP2 expression in the indicated spinal cord (n  =  4). (F) The BMS locomotion scores at different times after injury in the Sham, SCI, MM, MM‐NPs@NC, and MM‐NPs@PLK1 groups (n = 8). BMS, Basso Mouse Scale. (G) Representative CatWalk gait of the print view, timing view, area chart of print intensity, and foot view for each group (n = 6). (H) Regularity index (%) (n = 6), a measure of interlimb coordination based on the CatWalk gait data. (I) Representative recorded cortical motor evoked potentials (MEPs) and quantitative analysis of MEP amplitude (n = 5). The data are presented as the mean ± SD. ^*^p < 0.05, ^**^p < 0.01, ^****^p < 0.0001.

However, structural preservation does not necessarily translate into full functional restoration. We therefore systematically evaluated locomotor recovery using the Basso Mouse Scale (BMS) on days 1, 3, 7, 14, 21, and 28 post‐SCI (Figure 9F). Compared with those in the SCI group, the BMS scores in the MM‐NPs@PLK1 group were significantly higher. We further evaluated voluntary and coordinated motor functional recovery at 4 weeks post‐injury using CatWalk analysis. The MM‐NPs@PLK1 group exhibited more continuous footprint patterns and a significantly greater regularity index, indicating improved locomotor coordination (Figure 9G,H; Videos S1–S5). Finally, we measured motor evoked potentials (MEPs) to evaluate the recovery of motor signal conduction, a direct indicator of neural circuit integrity. Compared with those in the Sham group, MEP amplitudes in the SCI group were markedly lower at 4 weeks post‐injury, and the MM and MM‐NPs@NC groups showed similarly low amplitudes. In contrast, compared with those in the SCI, MM, and MM‐NPs@NC groups, the MEP amplitude in the MM‐NPs@PLK1 group was significantly higher, although the amplitude remained lower than that in the Sham group (Figure 9I). Taken together, the results of the BMS, CatWalk, and MEP analyses consistently support partial functional recovery following MM‐NPs@PLK1 treatment.

Moreover, to further validate the efficacy of MM‐NPs@PLK1 in a more severe injury model, we performed complete spinal cord transection at the T9 segment in mice. All mice in the Sham, SCI, MM, MM‐NPs@NC, and MM‐NPs@PLK1 groups were assessed 4 weeks post‐injury. H&E staining revealed significant chronic cavities and poor tissue integrity in the SCI, MM, and MM‐NPs@NC groups, whereas these features were attenuated in the MM‐NPs@PLK1 group (Figure S6A). To further characterize the lesion boundary in the complete spinal cord transection model, whole‐section immunofluorescence staining for GFAP, NF200, and MAP2 was performed at 28 days post‐injury. In the SCI, MM, and MM‐NPs@NC groups, a broad lesion gap remained clearly visible between the rostral and caudal spinal cord stumps, with dense GFAP‐positive astroglial borders and only sparse NF200‐ and MAP2‐positive structures within the lesion region. In the MM‐NPs@PLK1 group, the residual lesion gap appeared smaller, and stronger NF200 and MAP2 immunofluorescence signals were observed adjacent to the lesion; however, anatomical continuity across the transection site was not fully restored. (Figure S6B). These findings indicate that MM‐NPs@PLK1 treatment was associated with a reduced lesion gap and enhanced tissue remodelling without complete anatomical reconnection of the transected spinal cord. Compared with those in the SCI, MM, and MM‐NPs@NC groups, the BMS scores in the MM‐NPs@PLK1 group were significantly increased at 4 weeks post‐injury (Figure S6C). CatWalk analysis revealed a concordant increase in the regularity index, suggesting a partial improvement in locomotor coordination (Figure S6D,E).

## 3 Discussion

In this study, we identified PLK1 as a key regulator of macrophage efferocytosis following SCI and elucidated its role in promoting migrasome‐mediated mitocytosis. We further developed a biomimetic nanodelivery system (MM‐NPs@PLK1) that preferentially accumulates in injured spinal tissue, promotes macrophage‐associated PLK1 delivery, enhances mitocytosis and efferocytosis, and improves functional recovery.

SCI pathology presents a central dilemma: macrophage efferocytosis is essential for clearing ACs and resolving inflammation [40]. However, continual efferocytosis leads to the engulfment of a large number of ACs, imposing an immense degradation burden on the lysosomes (the cell's “digestive factory”) [13, 15]. Moreover, the mitochondrial damage induced by SCI also requires urgent repair. Cells typically rely on conventional MQC mechanisms, such as mitophagy, to maintain mitochondrial homeostasis, but critically, these conventional MQC pathways are heavily dependent on lysosomal degradation [41, 42, 43]. This creates a fatal conflict: within macrophages, mitochondrial repair (via mitophagy) and AC digestion (via efferocytosis) compete for and exhaust limited amounts of lysosomal resources. This renders the macrophage incapable of handling both tasks, leading to the dual collapse of efferocytosis function and mitochondrial homeostasis. Therefore, an alternative, lysosome‐independent MQC strategy is urgently needed in the context of SCI.

The core innovation of our study is the demonstration that migrasome‐mediated mitocytosis is the ideal solution to this conflict. Functionally, mitocytosis clears depolarized mitochondria to prevent MMP loss and stress‐induced respiratory chain dysfunction [21]. While mitocytosis does not differ much from other MQC strategies under physiological conditions, it has unique advantages under pathological conditions. In addition to preventing increases in the lysosomal burden, migrasomes rapidly encapsulate and expel damaged mitochondria, which is critical for maintaining efficient efferocytosis after SCI. Moreover, macrophage migration into damaged tissues facilitates mitocytosis [12], which is crucial for maintaining efficient efferocytosis after SCI [44]. Emerging evidence suggests that migrasome‐mediated mitocytosis functions as a selective MQC mechanism. Damaged mitochondria with elevated ROS levels and reduced membrane potential preferentially undergo peripheral trafficking and incorporation into migrasomes through KIF5B‐, Myo19‐, and Drp1‐dependent processes [21, 45]. In the present study, PLK1 overexpression reduced mitochondrial ROS accumulation while restoring mitochondrial membrane potential and promoting migrasome biogenesis, supporting a role for PLK1 in maintaining mitochondrial homeostasis. Thus, in SCI and other pathologies, mitocytosis offers a unique therapeutic strategy as an efficient, lysosome‐independent mechanism for clearing damaged mitochondria, thereby ensuring sustained efferocytosis.

Having established the critical role of mitocytosis, we further sought to identify the upstream molecules that regulate this process. Considering the key role of PLK1 in the cell cycle and mitochondrial function [46, 47], we explored its role in MQC. Previous studies have shown that PLK1 deficiency leads to excessive mitochondrial fragmentation and reduced ATP production [32], but its specific mechanism in MQC remains poorly characterized. Herein, via subcellular localization assays, we showed that PLK1 is partially localized to the outer mitochondrial membrane. This prompted us to explore the potential role of PLK1 in mediating MQC. Members of the tetraspanin family play crucial roles in migrasome formation [21, 24]. Our study revealed that PLK1 regulates TSPAN4‐related mitocytosis; this interplay between efferocytosis and mitocytosis is evidence of a PLK1‐mediated synergistic response that ensures efficient AC clearance and microenvironmental homeostasis restoration. This effect may mitigate inflammation and tissue damage and promote an environment conducive to healing. Future studies should elucidate the molecular pathways through which PLK1 promotes mitocytosis and efferocytosis to identify new targets for treating inflammatory and degenerative diseases.

To translate this mechanism into a therapy, we developed a biomimetic delivery strategy. Cell membrane‐coated nanoparticles have recently attracted significant attention because of their ability to mimic selected cell‐surface properties, improve biocompatibility, and prolong circulation [48, 49]. During secondary SCI, recruited macrophages accumulate within inflammatory lesions [44], and macrophage membrane‐coated nanoparticles preferentially accumulate at inflammatory sites through membrane‐associated homing interactions [50, 51]. In our system, macrophage membrane coating primarily promoted lesion accumulation and macrophage‐associated uptake, whereas ROS subsequently acted as an intracellular trigger for the dissociation of the CBP5/DNA nanocomplex and the release of the PLK1‐encoding plasmid. Consistent with this sequential design, MM‐NPs showed sustained accumulation in the injured spinal cord, and Cy5‐labelled DNA was detected within F4/80‐positive cells. Functionally, MM‐NPs@PLK1 enhanced mitocytosis‐ and efferocytosis‐related readouts, attenuated inflammation, supported the preservation of neural structure, and improved locomotor and electrophysiological outcomes. Importantly, these findings support macrophage‐associated delivery rather than absolute cell‐type exclusivity, and uptake by other cell populations within the complex SCI microenvironment cannot be completely excluded.

Despite the careful preparation of these experiments and the promising results, several limitations should be acknowledged. First, the present study employed MM‐NPs@PLK1 as a postinjury gain‐of‐function intervention designed to favor macrophage‐associated delivery. It does not establish that endogenous macrophage PLK1 is indispensable for mitocytosis, efferocytosis, or functional recovery after SCI. Future studies using carefully optimized inducible macrophage‐specific PLK1 knockout models and/or macrophage‐directed AAV approaches with appropriate spatial and temporal control will be valuable for independently defining the autonomous and necessary role of endogenous PLK1. Second, although macrophage membrane coating and the localization of Cy5‐labelled cargo within F4/80‐positive cells support macrophage‐associated delivery, absolute cellular exclusivity cannot be established, and uptake by other cell populations within the injured spinal cord cannot be completely excluded. Third, this study focused on mitocytosis, and whether PLK1 contributes to SCI improvement through other MQC pathways or mechanisms remains to be explored. Finally, although MM‐NPs@PLK1 showed favorable short‐term biosafety, its long‐term biodistribution, immunological effects, and biological safety require further investigation.

## 4 Conclusion

In conclusion, our study reveals a PLK1‐TSPAN4‐mitocytosis axis that supports macrophage efferocytosis by promoting the extrusion of damaged mitochondria through migrasomes and thereby provides a lysosome‐independent route for mitochondrial quality control. The biomimetic MM‐NPs@PLK1 nanoplatform enables injury‐responsive, macrophage‐associated PLK1 delivery and represents a promising strategy for modulating this pathway in SCI and potentially other inflammatory or degenerative conditions characterized by defective efferocytosis and mitochondrial dysfunction.

## 5 Experimental Section

### 5.1 Single‐Cell RNA Sequencing (scRNA‐seq) Data Acquisition and Processing

The scRNA‐seq data of both normal and injured spinal cord samples (n = 3) from wild‐type mice at 1, 3, and 7 days post‐injury (dpi) were sourced from the figshare database (https://doi.org/10.6084/m9.figshare.17702045). The raw scRNA sequencing data were processed using the Seurat package (version 4). Low‐quality cells were filtered out on the basis of standard quality control criteria: cells with fewer than 200 or more than 7000 detected genes, as well as those with mitochondrial gene content exceeding 10%, were removed to exclude damaged or dying cells. After filtering, the data from individual samples were normalized, scaled, and transformed. Harmony was applied to integrate samples and correct for batch effects across different time points and experimental batches. The data were subsequently used for dimensionality reduction, neighbourhood identification, and cluster analysis to explore the underlying cellular architecture. Cell clusters were annotated by referencing differentially expressed genes (DEGs) and known markers from previously published literature. The proportions of different cell types were visualized via uniform manifold approximation and projection (UMAP). For a deeper exploration of cell type functions, enrichment analysis of DEGs was conducted with the aid of the cluster Profiler package; this analysis was focused on Gene Ontology (GO) biological processes, and statistical significance was defined as an adjusted *p*‐value of <0.05.

### 5.2 RNA‐Seq Data and Analysis

Total RNA was extracted from BMMs (1 × 10^6^ per well) using TRIzol reagent (Takara) according to the manufacturer's instructions. Libraries were subsequently constructed using a VAHTS Universal V10 RNA‐seq Library Prep Kit (premixed version) according to the manufacturer's instructions. Transcriptome sequencing and analysis were conducted by OE Biotech Co., Ltd. (Shanghai, China). The libraries were subsequently sequenced on the Illumina NovaSeq 6000 platform, and 150 bp paired‐end reads were generated. A *q* value<0.05 and a fold change >2 or <0.5 were set as the thresholds for significant differential expression. On the basis of the hypergeometric distribution, GO enrichment analysis of the DEGs was performed to identify significantly enriched terms using R (v3.2.0). Additionally, the entire gene list ranked by log2 (fold change) was analyzed via GSEA (v4.3.2) with KEGG pathway sets.

### 5.3 Cell Culture

Fresh BMMs from mice were isolated as previously reported [52]. Briefly, after being euthanized under deep anaesthesia, 6–8‐week‐old mice were immersed in 75% ethanol, and their femurs and tibias were carefully excised. Muscles and connective tissues were completely removed. Bones were disinfected again with 75% ethanol and transferred to a sterile biosafety cabinet. Bone marrow from the tibia and femur was flushed and then cultured in α‐minimum essential medium (α‐MEM) supplemented with 10% FBS (Gibco, USA) and 1% penicillin‒streptomycin (complete α‐MEM). To obtain pure BMMs, nonadherent cells were collected and cultured in complete α‐MEM supplemented with M‐CSF (30 ng/mL; PeproTech, USA). The cells were cultured in this medium for 3 days, after which the medium was replaced. The cultures were continued for 5–7 days before the cells were used in the experiments. Jurkat cells and RAW264.7 cells were purchased from Procell. Jurkat cells were cultured in RPMI 1640 medium (Gibco, USA) supplemented with 10% foetal bovine serum and 1% penicillin G–streptomycin. RAW264.7 cells were cultured in high‐glucose DMEM (Gibco, USA) supplemented with 10% foetal bovine serum and 1% penicillin G–streptomycin. All the cells were cultured at 37°C in a humidified atmosphere containing 95% air and 5% carbon dioxide.

### 5.4 Transfection

BMMs were cultured in six‐well plates (5×10^5^ cells per well) and serum‐starved for 30 min before transfection. To induce overexpression, 2.5 µg of the PLK1 plasmid (Tsingke, China) or TSPAN4 plasmid (GenePharma, China) was transfected into BMMs, while cells in the control group were transfected with an empty vector (PLVX‐Puro plasmid vector; Table S1). In the siRNA experiments, 50 nm TSPAN4 siRNA (GenePharma, China) was transfected into the BMMs, and the cells in the control group were transfected with scrambled siRNA (GenePharma, China). For the complementary rescue experiments, PLK1 knockdown was performed using a PLK1‐targeting siRNA under the same siRNA transfection workflow, followed by TSPAN4 plasmid overexpression; a scrambled siRNA served as the negative control. Transfection was performed using Lipofectamine 3000 (Invitrogen, USA) according to the manufacturer's instructions. After transfection, the growth medium was replaced, and the cells were harvested after 24 h for RNA analysis and after 48 h for protein analysis. PLK1 or TSPAN4 expression was assessed by RT‒qPCR or Western blotting.

### 5.5 Single and Continual Efferocytosis Assays

BMMs were seeded in confocal dishes or six‐well plates and treated with H_2_O_2_ (150 µm) for 2 h. To induce apoptosis, Jurkat cells were irradiated with 254 nm ultraviolet light for 30 min and then incubated in PBS for 2 h. Apoptotic Jurkat cells were then stained with PKH26, PKH67 (Sigma‒Aldrich), or carboxyfluorescein diacetate succinimidyl ester (CFSE; Yeasen) according to the manufacturer's protocol. After thorough washing to remove unbound dye, the cells were resuspended in complete 1640 medium and added to BMMs at a 5:1 ratio of ACs: BMMs for the indicated durations. Efferocytosis was assessed by immunofluorescence, flow cytometry, or Incucyte. For the single efferocytosis assay, F4/80‐stained BMMs (red) were incubated with CFSE‐stained ACs (green) at a 5:1 ratio of ACs: BMMs for 45 min. After 45 min, the unengorged ACs were removed by repeated washes with PBS, after which the cells were visualized and imaged using a laser confocal microscope (Leica, Germany) for quantitative analysis by flow cytometry and Incucyte. For the continual efferocytosis assay, macrophages were first incubated with PKH67‐stained ACs (green) at a 5:1 ratio of ACs: BMMs for 45 min. Unbound ACs were then removed, and the cells were incubated in complete medium for 2 h. The macrophages were subsequently incubated with PKH26‐stained ACs (red) for 45 min at a 5:1 ratio of ACs: BMMs. After 45 min, the unbound ACs were removed, and fluorescence and bright‐field images were acquired using a laser confocal microscope (Leica, Germany) to quantify the number of BMMs with both green and red ACs relative to the number of BMMs with only green ACs as an indicator of continual efferocytosis.

### 5.6 In Vitro Immunofluorescence

For immunofluorescence staining, BMMs were seeded on glass‐bottom confocal dishes. After receiving the indicated treatments, the cells were washed with PBS and fixed with 4% paraformaldehyde (PFA) for 15 min at room temperature. Following fixation, the cells were permeabilized with 0.1% Triton X‐100 in PBS for 10 min. Nonspecific binding was blocked using 5% BSA for 1 h at room temperature. The cells were then incubated with primary antibodies (F4/80, TOM20, and PLK1) overnight at 4°C, followed by incubation with the corresponding Alexa Fluor 488‐ or 594‐conjugated secondary antibodies for 1 h at room temperature in the dark. For specific dye staining (e.g., PKH26, PKH67, CFSE, WGA, MitoTracker, and LysoTracker), cells were processed according to the specific protocols detailed in each assay section, often on live cells before fixation or according to the manufacturer's instructions. The nuclei were counterstained with Hoechst 33342 or DAPI for 15 min. Images were acquired using a laser confocal microscope (Leica, Germany) and analysed with ImageJ software.

### 5.7 Flow Cytometry

To assess efferocytosis efficiency, the treated cells were suspended in FACS staining buffer (PBS containing 2% FBS and 1 mM EDTA) at a density of 1 × 10^6^ cells/100 µL and incubated on ice for 30 min with an Fc blocker (anti‐mouse CD16/32; BioLegend) to block the nonspecific binding of immunoglobulins to Fc receptors. The cells were then incubated with fluorescent antibodies on ice for 60 min. The cells were subsequently washed twice in FACS buffer, resuspended, and analysed with a BD FACSCanto II flow cytometer. The data were analysed using FlowJo software 10.

### 5.8 Real‐time Efferocytosis Assay Using the IncuCyte Zoom and IncuCyte S3 Live‐Cell Analysis Systems

ACs were labelled with a pHrodo Red kit (Invitrogen) according to the manufacturer's instructions and added to each well of treated BMMs (ACs: BMMs = 5:1). The cell plates were analysed using the IncuCyte S3 Live Cell System to acquire real‐time images of the cells [53]. The instrument settings were those recommended by the manufacturer. Images were acquired every hour for 12 h. The intensity of red fluorescence produced after the phagocytosis of ACs (pHrodo Red‐labelled cells within the acidic microenvironment of macrophage lysosomes) is expressed as the total red object integrated intensity (Red Calibrated Unit [RCU]×µm^2^/image). The automated image analysis function of the Incucyte system was used for quantitative analysis of phagocytosis over time.

### 5.9 Phagolysosomal Acidification Assay

BMMs were seeded in confocal dishes and treated as indicated. To assess phagolysosomal acidification during efferocytosis, BMMs were cocultured with CFSE‐labelled ACs (green) (ACs: BMMs = 5:1) for 1, 2, or 3 h, as described for the efferocytosis assay. At the end of the coculture, the cells were washed with PBS to remove nonengulfed ACs. LysoTracker Deep Red (50 nm; Invitrogen) was then added to the live cells, which were then incubated at 37°C for 30 min to label the acidic compartments. The nuclei were counterstained with Hoechst 33342 (blue). Phagocytosis was then assessed by confocal imaging.

### 5.10 Real‐time Quantitative Polymerase Chain Reaction (RT‒qPCR)

Total RNA was isolated from cells using an RNA kit (Accurate Biology, China) according to the manufacturer's instructions, and the RNA was reverse transcribed into complementary DNA using a reverse transcription reagent (Accurate Biology, China). Real‐time quantitative PCR (RT‒qPCR) was performed using a qPCR kit and a Roch Real‐Time PCR Detection System according to the manufacturer's instructions. The primers used were purchased from Accurate Biology. All the primers used are listed in Table S2.

### 5.11 Western Blotting

Tissues and cells were lysed using buffer (RIPA; Beyotime, China) containing protease and phosphatase inhibitors to prevent degradation. The lysates were then centrifuged at 12 000 rpm for 10 min at 4°C, after which the supernatant was collected. The protein concentration in the supernatant was determined using a BCA assay (Beyotime, China). Polyacrylamide gels of appropriate concentrations were prepared on the basis of the size of the target protein. The samples were mixed with loading buffer, boiled, and electrophoresed at 120 V. The separated proteins were transferred to a PVDF membrane (Millipore, USA) by wet transfer. The membrane was blocked with 5% nonfat milk to prevent nonspecific binding. Primary antibodies were added for incubation overnight at 4°C, followed by three washes with Tris‐buffered saline supplemented with 0.1% Tween‐20 (TBST). The membranes were incubated with secondary antibodies for 1 h at room temperature and then washed with TBST. Finally, chemiluminescence reactions were performed using an ECL substrate, and the signals were captured and analysed using an imaging system.

### 5.12 Mitochondrial Function Evaluation

#### 5.12.1 Mitochondrial ROS Assay

MitoSOX (Invitrogen, USA), a fluorescent dye that specifically targets mitochondria, was used to measure mitochondrial ROS levels according to the manufacturer's instructions. Representative images were acquired and quantitatively analysed using a confocal microscope (Leica, Germany).

#### 5.12.2 Mitochondrial Membrane Potential Measurement

Mitochondrial membrane potential (MMP) was measured using the fluorescent probe JC‐1 (Beyotime, China). JC‐1 working solution was prepared according to the manufacturer's instructions. After pretreatment, the cells were washed three times with PBS and then incubated with JC‐1 working solution at 37°C in the dark for 20 min. The supernatant was discarded, and the cells were subsequently washed twice with JC‐1 staining solution. Changes in the ratio of red to green fluorescence reflect changes in the mitochondrial membrane potential. Representative images were acquired using a confocal microscope (Leica, Germany) and quantitatively analysed. The MMP was also monitored using IncuCyte real‑time live‑cell imaging in combination with tetramethylrhodamine methyl ester (TMRM; Invitrogen), a membrane‑potential‑sensitive fluorescent probe. TMRM was added to the culture medium at a final concentration of 40 nM. The TMRM fluorescence signal was quantified as RCU, which reflects the overall mitochondrial membrane potential level of the macrophages. Images were acquired every 15 min using the IncuCyte S3 system, and the RCU values were calculated by the IncuCyte software.

#### 5.12.3 ATP Level Measurement

ATP content in cells or tissues was determined using an ATP assay kit (Beyotime, China). Briefly, the samples were lysed, and the working solution was added to complete the reaction. The RLU values of the samples were then measured using a luminometer (Tecan, Switzerland). The ATP concentration in each sample was calculated using a standard curve.

### 5.13 Evaluation of Migrasome‐mediated Mitocytosis

To assess mitocytosis, BMMs were seeded at a density of 5 × 10^4^ cells per Petri dish and cultured for 24 h. BMMs subjected to various treatments were stimulated with H_2_O_2_ for 4 h. Afterward, the medium was replaced with fresh medium, the BMMs were labelled with WGA‐FITC, and the mitochondria were labelled with MitoTracker (Invitrogen, USA) for 15 min at 37°C. Afterward, the cells were rinsed with PBS, and the nuclei were stained with Hoechst 33342 for 15 min. Confocal microscopy was utilized for image capture.

### 5.14 Separation and Purification of Migrasomes

Migrasomes were isolated by iodixanol‐sucrose density gradient centrifugation using an OptiPrep kit (Sigma‒Aldrich, Germany) according to previously reported methods [54]. Briefly, BMMs were cultured in complete α‐MEM for 12 h in 150 mm dishes precoated with fibronectin (1 µg/ml). The growth medium was discarded, and the cells were washed to remove other extracellular vesicles from the medium. The cells were trypsinized and collected in 50 mL tubes. All subsequent manipulations were performed at 4°C. Cells and large debris were removed by centrifugation at 1000 × g for 10 min, followed by centrifugation at 4000 × g for 20 min. The crude migrasome pellet was then collected by centrifugation at 18 000 × g for 30 min. The supernatant containing the crude migrasome fraction was centrifuged at 150 000 × g for 4 h and fractionated using the following multistep OptiPrep dilution gradient: 2%, 5%, 8%, 10%, 12%, 15%, 19% (sample), 25%, and 30%. The resulting sample was suitable for Western blot analysis.

### 5.15 Molecular Docking Analysis

The three‐dimensional structures of the proteins were obtained from the UniProt database, and molecular docking analysis of PLK1 and TSPAN4 was performed using HDOCK. The docking results were visualized using PyMOL.

### 5.16 Coimmunoprecipitation (Co‐IP) Assay

Cells (1×10^7^) were collected and washed three times with precooled 1× PBS, after which 1 mL of cell lysis buffer and the appropriate amount of protein‐A/G agarose beads were added, and prebinding was performed at 4°C for 10–30 min. Next, the cell lysates (containing protease inhibitors) were divided into input, IP and IgG groups at a ratio of 1:4:2. The target antibody and IgG antibody were added to the IP and IgG groups, respectively, and the samples were incubated at 4°C for 12–18 h. Afterward, the samples were washed three times with precooled washing buffer, loading buffer was added, and the samples were boiled in boiling water for 10 min. After centrifugation, the supernatant was subjected to SDS‒PAGE as previously described, and PLK1 (Proteintech), TSPAN4 (ABclonal), and GAPDH (Proteintech) expression was analysed by Western blotting.

### 5.17 Preparation and Characterization of the NPs

CBP5 was synthesized as previously reported [33]. In brief, as depicted in the schematic (Scheme 1), compounds 1 and 2 were prepared according to previously described methods [55, 56]. For the synthesis of CBP5, 2‐(4‐(bromomethyl)phenyl)‐4,4,5,5‐tetramethyl‐1,3,2‐dioxaborolane (0.98 g, 3.30 mmol) was introduced to a solution of compound 2 (0.48 g, 0.30 mmol) dissolved in acetonitrile (50 mL). The resulting mixture was stirred under reflux for 24 h. The mixture was subsequently allowed to cool to ambient temperature and then concentrated via evaporation. An excess of ether was added to the concentrated solution to induce precipitation. The precipitate was isolated through filtration, and the solid was thoroughly washed with ice‐cold ether three times to eliminate any residual reactants. Finally, CBP5 was obtained as a white solid, with a yield of 87% (1.20 g), following vacuum drying (Scheme 1).

**SCHEME 1:**
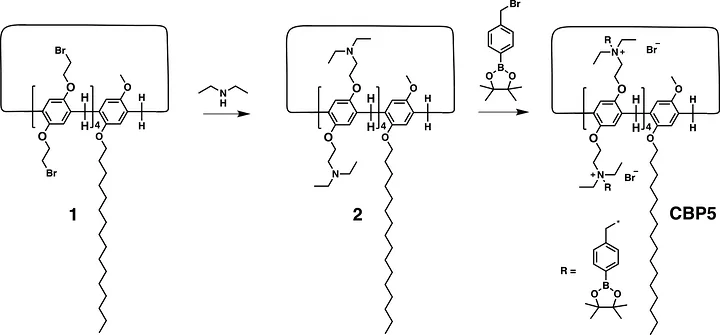
Synthetic route to CBP5.

In this study, plasmid DNA was dissolved in HEPES buffer (10 mM, pH 7.4) to a final concentration of 40 µg/mL. CBP5 was subsequently diluted in PBS to various concentrations corresponding to previously determined nitrogen‐to‐phosphorus (N/P) ratios of 10, 15, 20, 25, and 30. Equal volumes of the CBP5 solution were then added to the DNA mixture. The resulting mixtures were immediately vortexed for 20 s and subsequently incubated statically for 30 min to facilitate the formation of nanoparticles (NPs). The hydrodynamic diameters and zeta potentials of the NPs were characterized using dynamic light scattering (DLS) (Malvern, UK). The encapsulation efficiency of the NPs, as well as their responsiveness to ROS, was evaluated through gel retardation assays. Specifically, NPs with varying N/P ratios were treated with or without 5 mM H_2_O_2_ at 37°C for 1 h, followed by electrophoresis on a 1% agarose gel at 100 V for 30 min. Gel Red (Biotium) was employed as the DNA stain in the agarose gel to facilitate DNA detection. The morphology of the NPs was further examined by staining with water‐soluble phosphotungstic acid and visualization using a transmission electron microscope (JEM‐1400Plus).

### 5.18 Isolation of Macrophage Membranes

BMMs were isolated from 6‐ to 8‐week‐old C57BL/6J mice following an established protocol [57]. The isolation of macrophage membranes was performed as previously described [57, 58]. BMMs were suspended at a density of 5.0 × 10^6^ cells/ml in ice‐cold TM buffer (50 mM Tris‐HCl and 10 mM MgCl_2_, pH 7.5) and extruded through a miniextruder 40 times to disrupt cell integrity. Sucrose (1M ) was subsequently mixed with the cell homogenate to a final concentration of 0.25 M, and the mixture was centrifuged at 4°C and 2000 × *g* for approximately 10 min. The supernatant was then collected and subjected to further centrifugation at 3000 × g for 30 min. The resulting cell membranes were purified by rinsing with ice‐cold TM buffer containing 0.25 M sucrose. The total protein content in the purified membranes was quantified using a bicinchoninic acid (BCA) protein assay (Beyotime, China) according to the manufacturer's protocol. The membranes were stored at −80°C for future studies.

### 5.19 Preparation and Characterization of MMs and MM‐NPs

Following isolation, macrophage membranes were suspended in HEPES buffer at a concentration of 1 mg/ml and subsequently combined with an equal volume of nanoparticles (NPs). The mixture was then subjected to sonication for 5 min. Subsequent extrusion through 400, 200, and 100‐nm polycarbonate porous membranes was performed using an Avanti miniextruder (Xi'an Ruixi Biological Technology Co., Ltd., China) for at least 10 cycles to obtain macrophage membrane‐coated nanoparticles (MM‐NPs). MM vesicles were prepared by extruding the purified cell membranes through the same set of porous membranes. The sizes and zeta potentials of the MM and MM‐NPs were characterized by DLS. The morphological features of the MM and MM‐NPs were further examined with cryo–electron microscopy. A similar method was applied to prepare NPs@DNA^Cy5^, NPs@DNA^Plk1^, and ICG‐loaded MMs by replacing the NPs.

### 5.20 DNA Transfection Efficiency

To assess the efficiency of luciferase gene transfection, the pGL4.13 plasmid vector (Promega), which encodes the luciferase reporter gene, was used. BMMs were seeded at a density of 2 × 10^4^ cells per well in a 96‐well plate. The culture medium was then replaced with fresh medium containing either 0% or 10% foetal bovine serum (FBS). NP solutions (25 µL) with different N/P ratios were subsequently added to each well at a DNA dose of 1 µg per well, after which the cells were incubated for 4 h. Afterward, the medium was replaced with 200 µL of fresh complete medium, and the cells were cultured for an additional 44 h. The expression level of the luciferase plasmid was quantified in accordance with the manufacturer's protocol (Promega). The efficacy of luciferase gene transfection by nanoparticles (NPs) under oxidative conditions was evaluated. Cells were seeded in 96‐well plates at a density of 2 × 10^4^ cells per well and subsequently treated with varying concentrations of H_2_O_2_ (50, 100, 150, or 200 µm) for 6 h. Following pretreatment, NPs with an N/P ratio of 20 were added to each well, and the subsequent transfection efficiency was assessed. The protein concentration of the cell lysate was determined using a BCA protein assay kit. The luciferase activity was normalized to the protein concentration and expressed as relative luciferase luminescence units per milligram of protein.

### 5.21 Cellular Uptake Assay

The cellular uptake of the NPs by the BMMs was investigated using both confocal microscopy and flow cytometry. The DNA was labelled with Cy5 (Mirus Bio) in accordance with the manufacturer's protocol. The Cy5‐labelled DNA (DNA^Cy5^) was subsequently mixed with CBP5 at an N/P ratio of 20 prior to use. MM‐NPs were prepared as previously described. BMMs were seeded onto glass‐bottom Petri dishes at a density of 5 × 10^4^ cells per dish in 1.5 mL of cell culture medium. The medium was then replaced with fresh medium, and solutions (50 µL) of DNA^Cy5^, NPs@DNA^Cy5^, or MM‐NPs@DNA^Cy5^ were added at a DNA dose of 1 µg per dish. After 2 h of incubation, the nuclei were stained with Hoechst 33342 (Thermo Fisher Scientific, USA) at a concentration of two drops per millilitre of medium for 15 min. Flow cytometry was used to determine the percentage of Cy5‐positive cells, with 10 000 cells counted per treatment condition. A Nikon A1 system confocal fluorescence microscope (Japan) was used to obtain representative images.

### 5.22 MM and BMM Membrane Fusion

The MMs were labelled with 1,1′‐dioctadecyl‐3,3,3′,3′‐tetramethylindocarbocyanine perchlorate (DiI) for 15 min. MMs‐DiI were isolated by centrifugation at 10 000 × *g* for 10 min, after which the MMs were washed twice with PBS and resuspended in PBS at 1 mg/ml. BMMs were cultured in glass‐bottomed Petri dishes at a density of 5 × 10^4^ cells per dish as described above. BMMs were labelled with wheat germ agglutinin‐fluorescein isothiocyanate (WGA‐FITC) for 15 min. After being washed twice with PBS, the cells were incubated with complete medium. MM‐DiI solution was subsequently added to the medium containing the BMMs, and the mixture was incubated for 1 h. Images were acquired with a confocal fluorescence microscope (Nikon, A1, Japan). The fluorescence intensities of WGA‐FITC and MM‐DiI were analysed by ImageJ.

### 5.23 Effects of Efferocytosis Inhibitors on Cellular Uptake

BMMs were seeded in six‐well plates at a density of 2 × 10^5^ cells per well in 2 mL of culture medium and cultured overnight. The medium was subsequently replaced with 2 mL of fresh medium. Specific inhibitors were added to the medium to investigate their effects on cellular uptake mechanisms: chlorpromazine (a clathrin‐mediated endocytosis inhibitor), filipin III (a caveolae‐mediated endocytosis inhibitor), wortmannin (a phosphatidylinositol 3‐kinase‐mediated micropinocytosis inhibitor), and cytochalasin D (an actin polymerization inhibitor) at concentrations of 50, 7.5, 5, and 5 µm, respectively. After a 30‐min incubation, the NP and MM‐NP solutions were added to each well at 30 ng of DNA^Cy5^ per well, and the cells were cultured for an additional 2 h. Following incubation, the medium was removed, and the cells were washed with PBS, detached with trypsin, and isolated. The cells were then rinsed three times with PBS, resuspended in PBS, and immediately analysed using flow cytometry to determine the percentage of Cy5‐positive cells (1 × 10^4^ cells were counted per treatment).

### 5.24 Intracellular Trafficking of NPs in BMMs

To elucidate the ability of MM‐NPs to evade lysosomal degradation within BMMs, we employed a lysosomal marker for cellular staining. BMMs were seeded at a density of 5 × 10^4^ cells per Petri dish and cultured for 24 h. The culture medium was subsequently replaced with fresh medium containing DNA^Cy5^, NPs@DNA^Cy5^, or MM‐NPs@DNA^Cy5^ at a dose of 1 µg of DNA per dish. After 2 h of incubation, the lysosomes were labelled with LysoTracker Green (200 nm) for 15 min in accordance with the manufacturer's instructions, and the nuclei were stained with Hoechst 33342 for 15 min. Confocal microscopy was used for image capture. The fluorescence intensities of the lysosomes and DNA were analysed by ImageJ.

### 5.25 In Vivo Targeting Experiment

MM targeting was assessed using an IVIS Spectrum in vivo imaging system. ICG‐loaded MMs were prepared as described above. Before live‐animal imaging, the dorsal hair overlying the spinal cord region was removed to minimize optical interference. ICG or MM@ICG was injected into the tail vein of each mouse at a dose of 5 mg/kg in 200 µL. In vivo fluorescence images were acquired at 6, 24, and 72 h after injection using an excitation wavelength of 710 and a 785 nm filter. A fixed region of interest (ROI) centred on the injured spinal cord was applied consistently across the animals and time points, and the radiant efficiency was used for quantitative comparison. The intensity of the ROI, recorded as the mean of [(photons/sec/cm^2^/sr)/(µW/cm^2^)], was analysed to quantify the fluorescence intensity of ICG in the injured spinal cord.

To further evaluate the macrophage‐associated delivery of MM‐NPs, NPs@DNA^Cy5^ and MM‐NPs@DNA^Cy5^ were injected into mice via the tail vein at 3 days post‐injury. Spinal cord tissue sections were prepared to evaluate the in vivo distribution of the nanocomplexes. Briefly, spinal cord samples were harvested one day after injection and postfixed in 4% paraformaldehyde (PFA) for one day before being embedded in paraffin and sectioned. The sections were incubated with an anti‐F4/80 primary antibody overnight at 4°C. After primary antibody incubation, the sections were washed with PBS containing Tween 20 (PBST) three times for 7 min each and then incubated with the corresponding Alexa Fluor 488‐conjugated secondary antibody for 1 h at room temperature. The sections were rinsed three times with PBST and stained with DAPI Fluoromount‐G for 15 min. A confocal fluorescence microscope was used for image capture.

### 5.26 Animal Models

All animal experiments used were approved by the Institutional Animal Ethics Committee of Zhejiang University. The experiments were conducted in compliance with the Guide for the Care and Use of Laboratory Animals published by the National Institutes of Health. A standard mouse SCI model was established as previously described [59]. Briefly, 6–8‐week‐old C57BL/6 mice were divided into 5 groups: the Sham group, SCI group, MM group, MM‐NPs@NC group, and MM‐NPs@PLK1 group. The mice were anaesthetized with pentobarbital sodium (50 mg/kg, intraperitoneal injection) for surgical anaesthesia and analgesia. After the surgical area was shaved and disinfected, a midline skin incision was made to expose the dorsal spine, and the paravertebral muscles were gently retracted to expose the T9 vertebra. Laminectomy was then performed at T9 to expose the dorsal spinal cord without mechanical damage. Afterward, an impactor with a 1 mm diameter rod (RWD, China) was dropped at a speed of 3.5 m/s to induce contusion at a depth of 0.8 mm from the spinal cord surface, whereas the Sham group underwent laminectomy without contusion. In addition, we used a surgical blade to establish a complete spinal cord transection [60]. The incision was closed in layers, and the mice were placed on a warming pad for postoperative recovery. The criteria for successful induction of spinal cord injury were a tail‐flick response upon impact and complete paralysis of the hind limbs. To assist urination, manual bladder expression was performed twice daily until spontaneous bladder function was restored. The mice were administered tail‐vein injections every 2 days for 2 weeks. The sham and SCI groups received 100 µL of saline, while the treatment groups received 100 µL of 4.45 mg/kg nanoparticles per mouse (0.45 mg/kg plasmid DNA, 2.5 mg/kg membrane protein). Four weeks after surgery, all the mice were humanely sacrificed to evaluate the repair effects of MM‐NPs@PLK1.

### 5.27 Histological Analysis and Immunofluorescence Staining

Spinal cord tissue samples were collected 7 and 28 days after SCI. To ensure the biosafety of the treatment, major organs (heart, liver, spleen, lung, and kidney) were collected 28 days after injury. All tissue samples were fixed with 4% paraformaldehyde, dehydrated in a gradient manner, and embedded in paraffin. The embedded tissues were then cut into 5 µm‐thick sections and mounted on slides. Haematoxylin and eosin (H&E) staining: After the sections were dewaxed and rehydrated, they were stained with haematoxylin for 6 min to stain the cell nuclei. The sections were then differentiated with a 1% hydrochloric acid‐ethanol solution and blued with tap water. Afterward, the sections were stained with eosin for 2 min. Finally, the sections were dehydrated to clear them and mounted with neutral resin. For immunofluorescence staining, after dewaxing and hydration, the paraffin sections were thoroughly rinsed three times with phosphate‐buffered saline (PBS) for 5 min each to remove residual material and ensure proper tissue preparation. The sections were then boiled in citrate buffer for heat‐induced epitope retrieval. This process helps restore antigenicity that may have been masked during fixation. After they had cooled to room temperature, the sections were rinsed again with PBS three times for 5 min each to remove residual citrate buffer. To minimize nonspecific binding, the samples were blocked with 5% bovine serum albumin (BSA) solution. Next, the sections were incubated with the primary antibody overnight at 4°C to ensure specific binding to the target antigen. The sections were then washed three times with PBS and incubated with the corresponding Alexa Fluor 488‐ or Alexa Fluor 594‐conjugated secondary antibody and 1 mg/ml BODIPY for 1 h at room temperature. Finally, after three more washes with PBS, the sections were mounted with a fluorescence quencher containing DAPI for nuclei staining. The antibodies used are listed in Table S3.

### 5.28 Behavioral Assessments

Four weeks after SCI, motor recovery in the mice was assessed using the BMS and footprint analysis. The BMS was used to assess hindlimb behavior before surgery and weekly after spinal cord injury. The mice were allowed to walk freely on a grid and scored on the basis of hindlimb motor function, which ranged from 0 (complete paralysis) to 9 (normal movement). Gait was recorded and analysed using the CatWalk XT 10.6 system (Noldus, The Netherlands). Briefly, animals walked across a horizontal glass plate under fluorescent light. Footprints replaced the fluorescent light and were recorded by a camera located below the walkway. Raw data were collected and analysed using CatWalk 10.6 software. The software automatically marked the paw prints. The regularity index was selected for analysis. Five videos are shown, including Video S1 showing Sham, Video S2 showing SCI, Video S3 showing MM, Video S4 showing MM‐NPs@NC, and Video S5 showing MM‐NPs@PLK1. To ensure objective observation of behavioral recovery, two independent experimenters who were blinded to the experimental conditions assessed behavioral recovery.

### 5.29 Electrophysiological Assessment

Electrophysiological analysis was performed 4 weeks after surgery. Motor evoked potentials (MEPs) were recorded using an electromyograph and an evoked potential device (NIM‐Eclipse E4, Medtronic) as described previously [61]. All the mice received the same anaesthesia. Bipolar needle electrodes were placed directly on the skull to elicit MEPs. Recording needle electrodes were placed in the tibialis anterior muscles of both hindlimbs, and a needle electrode was inserted subcutaneously into the back as a ground. The peak amplitude of the MEP was recorded and compared across groups. Amplitude was defined as the maximum amplitude between the maximum positive and maximum negative peaks.

### 5.30 Biosafety Analysis

To evaluate safety, blood samples from Sham, SCI, MM, MM‐NPs@NC, and MM‐NPs@PLK1‐treated mice were collected for biochemical blood analyses. Moreover, the major organs (heart, liver, spleen, lungs, and kidneys) were collected and cut into 5 µm sections and then subjected to H&E staining.

### 5.31 Statistical Analysis

All the statistical analyses were performed using GraphPad Prism 10. All the data are presented as the mean ± standard deviation (SD). All the experiments were repeated at least three times. Comparisons between two independent groups were conducted via the two‐tailed unpaired Student's t‐test. When more than two groups with normally distributed datasets were compared, we performed one‐way analysis of variance (ANOVA) followed by Tukey's post hoc test or two‐way ANOVA with Šidák's multiple‐comparison test. The normality of the data distribution was assessed using the Shapiro‒Wilk test, and the homogeneity of variance for standard ANOVAs was evaluated using Levene's test. Data deviating from normality were analysed using the Mann‒Whitney U test (two groups) or the Kruskal‒Wallis test with Dunn's post hoc correction (more than two groups). For the BMS data collected over time, a two‐way repeated‐measures ANOVA with Šidák's multiple‐comparison test was applied. Sphericity was assessed using Mauchly's test, and the Greenhouse–Geisser correction was applied when the sphericity assumption was violated. Values of ^*^ *p* <0.05, ^**^ *p* <0.01, ^***^ *p* <0.001, and ^****^ *p* <0.0001 were considered to indicate statistical significance.

## Author Contributions

Z.H., Y.W., and Q.W. were responsible for conceptualization. Data curation was carried out by Z.H., J.H., J.Z., H.M., H.H., and N.L. The methodology was developed by Z.H., H.W., J.H., J.Z., H.T., and H.Z. Formal analysis was performed by Z.H., J.Z., J.H., H.M., H.T., Y.W., and P.Q. Supervision was provided by H.Z., D.H., and Q.W., who also managed Writing – review and editing alongside funding acquisition, which additionally included P.Q. and H.W. The original draft of the manuscript was written by Z.H., H.W., and J.H.

## Conflicts of Interest

The authors declare no conflicts of interest.

## Supporting information




**Supporting File 1**: advs78367‐sup‐0001‐SuppMat.docx.

**Supporting File 2**: advs78367‐sup‐0002‐SuppMat.zip.

## Data Availability

The data that support the findings of this study are available from the corresponding author upon reasonable request.
