# Supplementary material for: A Biomimetic PLK1 Nanodelivery System Promotes Migrasome‐Mediated Mitocytosis to Restore Efferocytosis and Functional Recovery After Spinal Cord Injury
Source: Adv Sci (Weinh). 2026 Oct 26:e78367. Online ahead of print. doi: 10.1002/advs.78367 (PMC13698012; doi:10.1002/advs.78367)
Supplement: Supplementary file 1 [file ADVS-9999-e78367-s001.docx]

Supporting Information

**A Biomimetic PLK1 Nanodelivery System Promotes Migrasome-Mediated Mitocytosis to Restore Efferocytosis and Functional Recovery after Spinal Cord Injury**

*Zhenxin Hu^1,2,3#^, Haoli Wang^1#^, Jing Huang^2#^, Junhua Zhou^2#^, Jialong Hou^4^, Hanwei Miao^2^, Huixin Tan^2^, Yining Wang^1^, Hao Hu^1^, Na Li^2^, Pengcheng Qiu^1^, Hongyu Zhang^2*^, Da He^5*^, Qingqing Wang^1,2*^*

^1^Department of Orthopaedic Surgery, Sir Run Run Shaw Hospital, Zhejiang University School of Medicine, Hangzhou, Zhejiang 310016, China.

^2^Cixi Biomedical Research Institute, Wenzhou Medical University, Ningbo, Zhejiang 315302, China.

^3^Department of Spine Surgery, Peking University Fourth School of Clinical Medicine, Beijing, 100035, China.

^4^Department of Neurology, The First Affiliated Hospital of Wenzhou Medical University, Wenzhou, Zhejiang 325000, China.

^5^Department of Spine Surgery, Beijing Jishuitan Hospital, Capital Medical University, Beijing, 100035, China.

^#^Zhenxin Hu, Haoli Wang, Jing Huang, and Junhua Zhou contributed equally to this work.

^*^Corresponding author. Email: st_hyz@126.com (H.Z.); heda@jst-hosp.com.cn (D.H.); wangqingqing@zju.edu.cn (Q.W.).

**Figure S1**

**Figure S1.** Single-cell RNA-sequencing analysis showing the temporal changes in relative PLK1 expression in macrophages in the Sham group and at 1, 3, and 7 days after SCI. PLK1 expression was normalized to the maximum value observed across the indicated time points (3 days), which was defined as 100%.

**Figure S2**

**Figure S2.** **Validation of PLK1 Expression in Different BMM Groups.** (A and B) Western blot analysis (A) and quantification (B) of PLK1 protein levels in the indicated BMM groups. (C) RT‒qPCR analysis of PLK1 mRNA levels (n = 3). The data are presented as the mean ± SD. **p < 0.01, ****p < 0.0001.

**Figure S3**

**Figure S3. PLK1 Promotes TSPAN4-mediated Mitocytosis.** (A-E) Western blot analysis (A) and quantification (B-E) of the expression of migrasome marker and mitochondrion-related proteins in the indicated migrasomes isolated from BMMs overexpressing PLK1 and TSPAN4 (n = 3). (F-J) Western blot analysis (F) and quantification (G-J) of migrasome marker and mitochondrion-related protein expression in isolated migrasome fractions from siPLK1+NC and siPLK1+OE-TSPAN4 BMMs (n = 3). (K) Representative immunofluorescence images of migrasomes (WGA-FITC, green) and mitochondria (MitoTracker, red) (n = 3). Scale bar = 20 μm. The data are presented as the mean ± SD. *p < 0.05, **p < 0.01.

**Figure S4**

**Figure S4. Characterization and Cellular Uptake Mechanism of MM-NPs@PLK1.** (A) Size and surface zeta potential of NPs@DNA at different N/P ratios. (B) Luciferase expression of NPs in RAW264.7 cells at a 20 N/P ratio after 0, 50, 100, 150, and 200 μM H_2_O_2_ treatment. (C) Cy5-positive BMMs were evaluated by flow cytometry after treatment with MM-NPs@DNACy5 for 2 hours. BMMs were cooled at 4°C or separately pretreated with endocytosis-related inhibitors (chlorpromazine, filipin III, wortmannin, and cytochalasin D) for 0.5 hours at 37°C. (D) MFI of Cy5 (n = 3). The data are presented as the mean ± SD. ****p < 0.0001.

**Figure S5**

**Figure S5. In Vivo Biosafety of MM-NPs@PLK1.** (A) H&E staining of heart, liver, spleen, kidney, and lung tissues from mice in the indicated groups to assess structural integrity and inflammatory cell infiltration. Scale bar = 200 μm. (B) Haematological and biochemical analyses, including WBC (white blood cell), RBC (red blood cell), HGB (haemoglobin), PLT (platelet), AST (aspartate aminotransferase), ALT (alanine aminotransferase), BUN (blood urea nitrogen‌), and UA (uric acid‌) analyses, in different groups at 4 weeks post-injury (n=5).

**Figure S6**

**Figure S6. MM-NPs@PLK1 Improves Motor Function Recovery in A Complete Spinal Cord Transection Model.** (A) Representative H&E staining images 28 days after SCI. Scale bar = 500 µm. (B) Representative triple immunofluorescence images of GFAP (red), NF200 (green), MAP2 (purple), and nuclei (DAPI, blue) staining in spinal cord tissues from different groups. Scale bar = 500 µm. Enlarged scale bar = 100 µm. (C) The BMS locomotion scores at different times after injury in different groups (n = 6). BMS, Basso Mouse Scale. (D) Representative CatWalk gait of the print view, timing view, area chart of print intensity, and foot view for each group. (E) Regularity index (%) (n = 6). The data are presented as the mean ± SD. **p < 0.01, ***p < 0.001, ****p < 0.0001.

**Supplementary Tables**

**Table S1 Plasmid Sequence**

| **Plasmid** | **Sequence** |
| --- | --- |
| PLK1 | GAATTCATGAATGCAGCGGCCAAAGCTGGAAAGCTGGCTCGAGCACCAGCCGACCTCGGGAAAGGTGGGGTTCCGGGAGATGCAGTTCCCGGTGCCCCAGTGGCCGCCCCGCTGGCGAAAGAAATTCCGGAGGTCCTAGTGGACCCACGCAGCCGGCGGCAGTATGTACGGGGGCGCTTTCTGGGTAAAGGAGGCTTCGCCAAATGCTTCGAGATCTCAGACGCAGACACAAAAGAGGTGTTCGCAGGCAAGATCGTGCCTAAGTCTTTGCTGCTCAAGCCCCACCAGAAGGAGAAGATGTCTATGGAGATCTCAATTCACCGCAGCCTAGCACACCAACACGTCGTAGGCTTCCATGACTTTTTTGAGGACAGCGACTTTGTATTTGTAGTTTTGGAGCTCTGTCGCAGGAGGTCCCTCCTGGAGCTGCACAAGAGGAGGAAGGCACTGACCGAGCCTGAGGCCCGCTACTACCTGCGACAGATAGTCCTGGGCTGCCAGTACCTGCACCGCAATCAGGTCATTCACAGGGACCTCAAGCTGGGCAACCTCTTCCTGAACGAGGATCTGGAGGTGAAAATAGGGGATTTTGGCTTGGCAACCAAAGTGGAATATGAAGGGGAACGAAAGAAGACCTTGTGTGGCACTCCTAACTACATAGCTCCTGAGGTGCTGAGCAAGAAGGGACACAGTTTTGAGGTGGATGTGTGGTCCATTGGGTGCATCATGTATACCTTGCTAGTGGGCAAGCCTCCCTTTGAGACCTCGTGCCTAAAAGAGACCTACCTCCGGATCAAGAAAAATGAATACAGTATTCCCAAGCACATCAACCCAGTGGCCGCCTCCCTCATCCAGAAGATGCTTCAGACAGACCCCACTGCCCGCCCCACCATTCACGAGTTGCTCAATGACGAGTTCTTCACTTCTGGCTACATCCCCGCCCGTCTCCCTATTACCTGCCTCACCATCCCACCAAGGTTTTCAATCGCTCCCAGCAGCCTGGACCCCAGCAGCAGGAAACCTCTCAAAGTCCTCAATAAAGGTGTGGAGAACCCCCTGCCTGACCGTCCCCGGGAGAAAGAGGAACCGGTGGTCCGGGAGACAAATGAGGCCATTGAGTGCCACCTTAGTGACTTGCTACAGCAGCTGACCAGTGTCAACGCCTCCAAGCCCTCGGAGCGCGGGCTGGTGCGGCAAGAGGAGGCTGAGGATCCTGCCTGCATCCCCATCTTCTGGGTCAGCAAGTGGGTGGACTATTCGGACAAGTATGGCCTTGGGTATCAGCTGTGTGACAACAGTGTGGGGGTGCTTTTTAATGACTCAACACGCCTGATTCTCTACAATGACGGGGACAGCCTGCAGTACATAGAGCGTGATGGCACGGAGTCCTATCTCACTGTGAGCTCCCATCCCAATTCCTTGATGAAGAAGATCACTCTCCTCAACTATTTCCGCAATTACATGAGTGAGCACCTGCTGAAGGCAGGGGCCAACATCACACCCCGGGAAGGCGACGAGCTGGCCCGGCTGCCCTACCTACGAACGTGGTTCCGCACACGCAGCGCCATCATCCTGCACCTCAGCAACGGCACCGTGCAGATTAACTTCTTCCAGGACCACACCAAACTTATCCTGTGCCCCCTGATGGCAGCGGTGACCTACATCAACGAGAAGAGGGACTTCCAAACGTACCGCCTGAGCCTCCTGGAGGAGTATGGCTGCTGCAAGGAGCTGGCCAGCCGCCTCCGCTACGCCCGCACCATGGTAGACAAGCTGCTGAGCTCGCGCTCCGCCAGCAACCGCCTCAAGGCCTCCTAGTCTAGA |
| TSPAN4 | ATGGCGCGCGGCTGCCTCCAGGGCGTCAAGTACCTCATGTTCGCCTTCAACCTGCTCTTCTGGCTGGGTGGCTGTGGTGTCCTGGGTGTTGGCATCTGGTTGGCTGCCACACAGGGAAACTTTGCCACCTTATCATCCTCATTTCCATCCTTGTCGGCTGCCAACCTGCTCATCGTCACCGGGACCTTCGTCATGGCCATCGGCTTCGTGGGCTGCATTGGGGCCCTCAAGGAGAACAAGTGCCTACTGCTCACTTTCTTTGTGCTGCTGCTGCTAGTGTTCCTGCTGGAAGCCACCATTGCTGTGCTCTTCTTTGCCTACAGTGACAAGATTGACAGTTATGCCCAACAAGACCTGAAGAAGGGCCTGCATCTGTATGGCACACAGGGCAACGTGGGCCTCACCAATGCCTGGAGCATCATCCAGACTGATTTCCGATGCTGTGGAGTTTCCAATTACACTGATTGGTTTGAGGTATACAATGCCACTCGTGTGCCTGACTCCTGCTGTCTGGAGTTCAGTGATAGCTGTGGGTTACATGAACCTGGTACCTGGTGGAAGTCGCCCTGTTATGAGACAGTGAAGGCCTGGCTCCAGGAGAACCTGCTAGCTGTGGGCATCTTTGGACTGTGCACGGCACTGGTGCAGATTCTGGGCCTCACCTTCGCTATGACCATGTACTGCCAGGTGGTAAAGGCGGACACCTACTGTGCATAG |
| plasmid vehicle (PLVX-Puro) | GGAAGGGCTAATTCACTCCCAAAGAAGACAAGATATCCTTGATCTGTGGATCTACCACACACAAGGCTACTTCCCTGATTAGCAGAACTACACACCAGGGCCAGGGGTCAGATATCCACTGACCTTTGGATGGTGCTACAAGCTAGTACCAGTTGAGCCAGATAAGGTAGAAGAGGCCAATAAAGGAGAGAACACCAGCTTGTTACACCCTGTGAGCCTGCATGGGATGGATGACCCGGAGAGAGAAGTGTTAGAGTGGAGGTTTGACAGCCGCCTAGCATTTCATCACGTGGCCCGAGAGCTGCATCCGGAGTACTTCAAGAACTGCTGATATCGAGCTTGCTACAAGGGACTTTCCGCTGGGGACTTTCCAGGGAGGCGTGGCCTGGGCGGGACTGGGGAGTGGCGAGCCCTCAGATCCTGCATATAAGCAGCTGCTTTTTGCCTGTACTGGGTCTCTCTGGTTAGACCAGATCTGAGCCTGGGAGCTCTCTGGCTAACTAGGGAACCCACTGCTTAAGCCTCAATAAAGCTTGCCTTGAGTGCTTCAAGTAGTGTGTGCCCGTCTGTTGTGTGACTCTGGTAACTAGAGATCCCTCAGACCCTTTTAGTCAGTGTGGAAAATCTCTAGCAGTGGCGCCCGAACAGGGACTTGAAAGCGAAAGGGAAACCAGAGGAGCTCTCTCGACGCAGGACTCGGCTTGCTGAAGCGCGCACGGCAAGAGGCGAGGGGCGGCGACTGGTGAGTACGCCAAAAATTTTGACTAGCGGAGGCTAGAAGGAGAGAGATGGGTGCGAGAGCGTCAGTATTAAGCGGGGGAGAATTAGATCGCGATGGGAAAAAATTCGGTTAAGGCCAGGGGGAAAGAAAAAATATAAATTAAAACATATAGTATGGGCAAGCAGGGAGCTAGAACGATTCGCAGTTAATCCTGGCCTGTTAGAAACATCAGAAGGCTGTAGACAAATACTGGGACAGCTACAACCATCCCTTCAGACAGGATCAGAAGAACTTAGATCATTATATAATACAGTAGCAACCCTCTATTGTGTGCATCAAAGGATAGAGATAAAAGACACCAAGGAAGCTTTAGACAAGATAGAGGAAGAGCAAAACAAAAGTAAGACCACCGCACAGCAAGCGGCCGGCCGCTGATCTTCAGACCTGGAGGAGGAGATATGAGGGACAATTGGAGAAGTGAATTATATAAATATAAAGTAGTAAAAATTGAACCATTAGGAGTAGCACCCACCAAGGCAAAGAGAAGAGTGGTGCAGAGAGAAAAAAGAGCAGTGGGAATAGGAGCTTTGTTCCTTGGGTTCTTGGGAGCAGCAGGAAGCACTATGGGCGCAGCGTCAATGACGCTGACGGTACAGGCCAGACAATTATTGTCTGGTATAGTGCAGCAGCAGAACAATTTGCTGAGGGCTATTGAGGCGCAACAGCATCTGTTGCAACTCACAGTCTGGGGCATCAAGCAGCTCCAGGCAAGAATCCTGGCTGTGGAAAGATACCTAAAGGATCAACAGCTCCTGGGGATTTGGGGTTGCTCTGGAAAACTCATTTGCACCACTGCTGTGCCTTGGAATGCTAGTTGGAGTAATAAATCTCTGGAACAGATTTGGAATCACACGACCTGGATGGAGTGGGACAGAGAAATTAACAATTACACAAGCTTAATACACTCCTTAATTGAAGAATCGCAAAACCAGCAAGAAAAGAATGAACAAGAATTATTGGAATTAGATAAATGGGCAAGTTTGTGGAATTGGTTTAACATAACAAATTGGCTGTGGTATATAAAATTATTCATAATGATAGTAGGAGGCTTGGTAGGTTTAAGAATAGTTTTTGCTGTACTTTCTATAGTGAATAGAGTTAGGCAGGGATATTCACCATTATCGTTTCAGACCCACCTCCCAACCCCGAGGGGACCCGACAGGCCCGAAGGAATAGAAGAAGAAGGTGGAGAGAGAGACAGAGACAGATCCATTCGATTAGTGAACGGATCTCGACGGTATCGCCTTTAAAAGAAAAGGGGGGATTGGGGGGTACAGTGCAGGGGAAAGAATAGTAGACATAATAGCAACAGACATACAAACTAAAGAATTACAAAAACAAATTACAAAAATTCAAAATTTTCGGGTTTATTACAGGGACAGCAGAGATCCAGTTTATCGATAAGCTTGGGAGTTCCGCGTTACATAACTTACGGTAAATGGCCCGCCTGGCTGACCGCCCAACGACCCCCGCCCATTGACGTCAATAATGACGTATGTTCCCATAGTAACGCCAATAGGGACTTTCCATTGACGTCAATGGGTGGAGTATTTACGGTAAACTGCCCACTTGGCAGTACATCAAGTGTATCATATGCCAAGTACGCCCCCTATTGACGTCAATGACGGTAAATGGCCCGCCTGGCATTATGCCCAGTACATGACCTTATGGGACTTTCCTACTTGGCAGTACATCTACGTATTAGTCATCGCTATTACCATGGTGATGCGGTTTTGGCAGTACATCAATGGGCGTGGATAGCGGTTTGACTCACGGGGATTTCCAAGTCTCCACCCCATTGACGTCAATGGGAGTTTGTTTTGGCACCAAAATCAACGGGACTTTCCAAAATGTCGTAACAACTCCGCCCCATTGACGCAAATGGGCGGTAGGCGTGTACGGTGGGAGGTCTATATAAGCAGAGCTCGTTTAGTGAACCGTCAGATCGCCTGGAGACGCCATCCACGCTGTTTTGACCTCCATAGAAGACACCGACTCTACTAGAGGATCGCTAGCGCTACCGGACTCAGATCTCGAGCTCAAGCTTCGAATTCTGCAGTCGACGGTACCGCGGGCCCGGGATCCCGCGACTCTAGATAATTCTACCGGGTAGGGGAGGCGCTTTTCCCAAGGCAGTCTGGAGCATGCGCTTTAGCAGCCCCGCTGGGCACTTGGCGCTACACAAGTGGCCTCTGGCCTCGCACACATTCCACATCCACCGGTAGGCGCCAACCGGCTCCGTTCTTTGGTGGCCCCTTCGCGCCACCTTCTACTCCTCCCCTAGTCAGGAAGTTCCCCCCCGCCCCGCAGCTCGCGTCGTGCAGGACGTGACAAATGGAAGTAGCACGTCTCACTAGTCTCGTGCAGATGGACAGCACCGCTGAGCAATGGAAGCGGGTAGGCCTTTGGGGCAGCGGCCAATAGCAGCTTTGCTCCTTCGCTTTCTGGGCTCAGAGGCTGGGAAGGGGTGGGTCCGGGGGCGGGCTCAGGGGCGGGCTCAGGGGCGGGGCGGGCGCCCGAAGGTCCTCCGGAGGCCCGGCATTCTGCACGCTTCAAAAGCGCACGTCTGCCGCGCTGTTCTCCTCTTCCTCATCTCCGGGCCTTTCGACCTGCAGCCCAAGCTTACCATGACCGAGTACAAGCCCACGGTGCGCCTCGCCACCCGCGACGACGTCCCCAGGGCCGTACGCACCCTCGCCGCCGCGTTCGCCGACTACCCCGCCACGCGCCACACCGTCGATCCGGACCGCCACATCGAGCGGGTCACCGAGCTGCAAGAACTCTTCCTCACGCGCGTCGGGCTCGACATCGGCAAGGTGTGGGTCGCGGACGACGGCGCCGCGGTGGCGGTCTGGACCACGCCGGAGAGCGTCGAAGCGGGGGCGGTGTTCGCCGAGATCGGCCCGCGCATGGCCGAGTTGAGCGGTTCCCGGCTGGCCGCGCAGCAACAGATGGAAGGCCTCCTGGCGCCGCACCGGCCCAAGGAGCCCGCGTGGTTCCTGGCCACCGTCGGCGTCTCGCCCGACCACCAGGGCAAGGGTCTGGGCAGCGCCGTCGTGCTCCCCGGAGTGGAGGCGGCCGAGCGCGCCGGGGTGCCCGCCTTCCTGGAGACCTCCGCGCCCCGCAACCTCCCCTTCTACGAGCGGCTCGGCTTCACCGTCACCGCCGACGTCGAGGTGCCCGAAGGACCGCGCACCTGGTGCATGACCCGCAAGCCCGGTGCCTGACCGCGTCTGGAACAATCAACCTCTGGATTACAAAATTTGTGAAAGATTGACTGGTATTCTTAACTATGTTGCTCCTTTTACGCTATGTGGATACGCTGCTTTAATGCCTTTGTATCATGCTATTGCTTCCCGTATGGCTTTCATTTTCTCCTCCTTGTATAAATCCTGGTTGCTGTCTCTTTATGAGGAGTTGTGGCCCGTTGTCAGGCAACGTGGCGTGGTGTGCACTGTGTTTGCTGACGCAACCCCCACTGGTTGGGGCATTGCCACCACCTGTCAGCTCCTTTCCGGGACTTTCGCTTTCCCCCTCCCTATTGCCACGGCGGAACTCATCGCCGCCTGCCTTGCCCGCTGCTGGACAGGGGCTCGGCTGTTGGGCACTGACAATTCCGTGGTGTTGTCGGGGAAGCTGACGTCCTTTCCATGGCTGCTCGCCTGTGTTGCCACCTGGATTCTGCGCGGGACGTCCTTCTGCTACGTCCCTTCGGCCCTCAATCCAGCGGACCTTCCTTCCCGCGGCCTGCTGCCGGCTCTGCGGCCTCTTCCGCGTCTTCGCCTTCGCCCTCAGACGAGTCGGATCTCCCTTTGGGCCGCCTCCCCGCCTGGAATTAATTCTGCAGTCGAGACCTAGAAAAACATGGAGCAATCACAAGTAGCAATACAGCAGCTACCAATGCTGATTGTGCCTGGCTAGAAGCACAAGAGGAGGAGGAGGTGGGTTTTCCAGTCACACCTCAGGTACCTTTAAGACCAATGACTTACAAGGCAGCTGTAGATCTTAGCCACTTTTTAAAAGAAAAGAGGGGACTGGAAGGGCTAATTCACTCCCAACGAAGACAAGATATCCTTGATCTGTGGATCTACCACACACAAGGCTACTTCCCTGATTAGCAGAACTACACACCAGGGCCAGGGGTCAGATATCCACTGACCTTTGGATGGTGCTACAAGCTAGTACCAGTTGAGCCAGATAAGGTAGAAGAGGCCAATAAAGGAGAGAACACCAGCTTGTTACACCCTGTGAGCCTGCATGGGATGGATGACCCGGAGAGAGAAGTGTTAGAGTGGAGGTTTGACAGCCGCCTAGCATTTCATCACGTGGCCCGAGAGCTGCATCCGGAGTACTTCAAGAACTGCTGATATCGAGCTTGCTACAAGGGACTTTCCGCTGGGGACTTTCCAGGGAGGCGTGGCCTGGGCGGGACTGGGGAGTGGCGAGCCCTCAGATCCTGCATATAAGCAGCTGCTTTTTGCCTGTACTGGGTCTCTCTGGTTAGACCAGATCTGAGCCTGGGAGCTCTCTGGCTAACTAGGGAACCCACTGCTTAAGCCTCAATAAAGCTTGCCTTGAGTGCTTCAAGTAGTGTGTGCCCGTCTGTTGTGTGACTCTGGTAACTAGAGATCCCTCAGACCCTTTTAGTCAGTGTGGAAAATCTCTAGCAGTAGTAGTTCATGTCATCTTATTATTCAGTATTTATAACTTGCAAAGAAATGAATATCAGAGAGTGAGAGGCCTTGACATTGCTAGCGTTTTACCGTCGACCTCTAGCTAGAGCTTGGCGTAATCATGGTCATAGCTGTTTCCTGTGTGAAATTGTTATCCGCTCACAATTCCACACAACATACGAGCCGGAAGCATAAAGTGTAAAGCCTGGGGTGCCTAATGAGTGAGCTAACTCACATTAATTGCGTTGCGCTCACTGCCCGCTTTCCAGTCGGGAAACCTGTCGTGCCAGCTGCATTAATGAATCGGCCAACGCGCGGGGAGAGGCGGTTTGCGTATTGGGCGCTCTTCCGCTTCCTCGCTCACTGACTCGCTGCGCTCGGTCGTTCGGCTGCGGCGAGCGGTATCAGCTCACTCAAAGGCGGTAATACGGTTATCCACAGAATCAGGGGATAACGCAGGAAAGAACATGTGAGCAAAAGGCCAGCAAAAGGCCAGGAACCGTAAAAAGGCCGCGTTGCTGGCGTTTTTCCATAGGCTCCGCCCCCCTGACGAGCATCACAAAAATCGACGCTCAAGTCAGAGGTGGCGAAACCCGACAGGACTATAAAGATACCAGGCGTTTCCCCCTGGAAGCTCCCTCGTGCGCTCTCCTGTTCCGACCCTGCCGCTTACCGGATACCTGTCCGCCTTTCTCCCTTCGGGAAGCGTGGCGCTTTCTCATAGCTCACGCTGTAGGTATCTCAGTTCGGTGTAGGTCGTTCGCTCCAAGCTGGGCTGTGTGCACGAACCCCCCGTTCAGCCCGACCGCTGCGCCTTATCCGGTAACTATCGTCTTGAGTCCAACCCGGTAAGACACGACTTATCGCCACTGGCAGCAGCCACTGGTAACAGGATTAGCAGAGCGAGGTATGTAGGCGGTGCTACAGAGTTCTTGAAGTGGTGGCCTAACTACGGCTACACTAGAAGAACAGTATTTGGTATCTGCGCTCTGCTGAAGCCAGTTACCTTCGGAAAAAGAGTTGGTAGCTCTTGATCCGGCAAACAAACCACCGCTGGTAGCGGTGGTTTTTTTGTTTGCAAGCAGCAGATTACGCGCAGAAAAAAAGGATCTCAAGAAGATCCTTTGATCTTTTCTACGGGGTCTGACGCTCAGTGGAACGAAAACTCACGTTAAGGGATTTTGGTCATGAGATTATCAAAAAGGATCTTCACCTAGATCCTTTTAAATTAAAAATGAAGTTTTAAATCAATCTAAAGTATATATGAGTAAACTTGGTCTGACAGTTACCAATGCTTAATCAGTGAGGCACCTATCTCAGCGATCTGTCTATTTCGTTCATCCATAGTTGCCTGACTCCCCGTCGTGTAGATAACTACGATACGGGAGGGCTTACCATCTGGCCCCAGTGCTGCAATGATACCGCGAGACCCACGCTCACCGGCTCCAGATTTATCAGCAATAAACCAGCCAGCCGGAAGGGCCGAGCGCAGAAGTGGTCCTGCAACTTTATCCGCCTCCATCCAGTCTATTAATTGTTGCCGGGAAGCTAGAGTAAGTAGTTCGCCAGTTAATAGTTTGCGCAACGTTGTTGCCATTGCTACAGGCATCGTGGTGTCACGCTCGTCGTTTGGTATGGCTTCATTCAGCTCCGGTTCCCAACGATCAAGGCGAGTTACATGATCCCCCATGTTGTGCAAAAAAGCGGTTAGCTCCTTCGGTCCTCCGATCGTTGTCAGAAGTAAGTTGGCCGCAGTGTTATCACTCATGGTTATGGCAGCACTGCATAATTCTCTTACTGTCATGCCATCCGTAAGATGCTTTTCTGTGACTGGTGAGTACTCAACCAAGTCATTCTGAGAATAGTGTATGCGGCGACCGAGTTGCTCTTGCCCGGCGTCAATACGGGATAATACCGCGCCACATAGCAGAACTTTAAAAGTGCTCATCATTGGAAAACGTTCTTCGGGGCGAAAACTCTCAAGGATCTTACCGCTGTTGAGATCCAGTTCGATGTAACCCACTCGTGCACCCAACTGATCTTCAGCATCTTTTACTTTCACCAGCGTTTCTGGGTGAGCAAAAACAGGAAGGCAAAATGCCGCAAAAAAGGGAATAAGGGCGACACGGAAATGTTGAATACTCATACTCTTCCTTTTTCAATATTATTGAAGCATTTATCAGGGTTATTGTCTCATGAGCGGATACATATTTGAATGTATTTAGAAAAATAAACAAATAGGGGTTCCGCGCACATTTCCCCGAAAAGTGCCACCTGACGTCGACGGATCGGGAGATCAACTTGTTTATTGCAGCTTATAATGGTTACAAATAAAGCAATAGCATCACAAATTTCACAAATAAAGCATTTTTTTCACTGCATTCTAGTTGTGGTTTGTCCAAACTCATCAATGTATCTTATCATGTCTGGATCAACTGGATAACTCAAGCTAACCAAAATCATCCCAAACTTCCCACCCCATACCCTATTACCACTGCCAATTACCTGTGGTTTCATTTACTCTAAACCTGTGATTCCTCTGAATTATTTTCATTTTAAAGAAATTGTATTTGTTAAATATGTACTACAAACTTAGTAGTT |
|  |  |

**Table S2 RT‒qPCR Primers**

| **Name** | **Forward (5’ to 3’)** | **Reverse (5’ to 3’)** |
| --- | --- | --- |
| iNOS | ACTACTGCTGGTGGTGACAA | GAAGGTGTGGTTGAGTTCTCTAAG |
| Il1β | TGGTGTGTGACGTTCCCATT | TGTCGTTGCTTGGTTCTCCT |
| Il6 | CCACTTCACAAGTCGGAGGCTTA | TGCAAGTGCATCATCGTTGTTC |
| Il10 | GCCAGAGCCACATGCTCCTA | GATAAGGCTTGGCAACCCAAGTAA |
| Ym1 | GATGGCCTCAACCTGGACTG | CGTCAATGATTCCTGCTCCTG |
| Plk1 | GGTTTTCAATCGCTCCCAGC | AGGGGGTTCTCCACACCTTT |
| Gapdh | AAATGGTGAAGGTCGGTGTGAAC | CAACAATCTCCACTTTGCCACTG |

**Table S3 Antibodies Used for Western Blotting or Immunofluorescence Staining**

| Antibody | Resource |
| --- | --- |
| PLK1 | Proteintech |
| MAC2 | Proteintech |
| F4/80 | Abcam |
| TOM20 | Proteintech |
| TIM23 | Proteintech |
| HSP60 | Proteintech |
| TSPAN4 | ABclonal |
| NF200 | Proteintech |
| MAP2 | Proteintech |
| GFAP | Abcam |
| CD11b | Abcam |
| β-actin | Proteintech |

**Supplementary Videos**

**Supplementary Video 1.** Dynamic Video of Motor Function Recovery in Mice, Related to Figure 9H (Sham)

**Supplementary Video 2.** Dynamic Video of Motor Function Recovery in Mice, Related to Figure 9H (SCI)

**Supplementary Video 3.** Dynamic Video of Motor Function Recovery in Mice, Related to Figure 9H (MM)

**Supplementary Video 4.** Dynamic Video of Motor Function Recovery in Mice, Related to Figure 9H (MM-NPs@NC)

**Supplementary Video 5.** Dynamic Video of Motor Function Recovery in Mice, Related to Figure 9H (MM-NPs@PLK1)
